# Transfer learning with fuzzy decision support for multi-class lung disease classification: performance analysis of pre-trained CNN models

**DOI:** 10.1038/s41598-025-19114-3

**Published:** 2025-10-08

**Authors:** Nand Lal Yadav, Sudhakar Singh, Rajesh Kumar, D. K. Nishad

**Affiliations:** 1 Department of Electronics and Communication, University of Allahabad, Prayagraj, India; 2 IBM Multi Activities Co. Ltd. Khartoum, Khartoum, Sudan; 3 Department of Electrical Engineering, Dr. Shakuntala Misra National Rehabilitation University, Lucknow, India

**Keywords:** Transfer learning, Convolutional neural networks, Fuzzy logic, Medical image analysis, Lung disease classification, Deep learning, Computer-aided diagnosis, Energy science and technology, Engineering

## Abstract

Accurate and efficient classification of lung diseases from medical images remains a significant challenge in computer-aided diagnosis systems. This research presents a novel approach integrating transfer learning techniques with fuzzy decision support systems for multi-class lung disease classification. We compare the performance of three pre-trained CNN architectures—VGG16, VGG19, and ResNet50—enhanced with a fuzzy logic decision layer. The proposed methodology employs transfer learning to leverage knowledge from large-scale datasets while adapting to the specific characteristics of lung disease images. A k-symbol Lerch transcendent function is implemented for image enhancement during preprocessing, significantly improving feature extraction capabilities by 23.4% in contrast enhancement and 18.7% in feature visibility. The fuzzy decision support system addresses inherent uncertainties in medical image classification through membership functions and rule-based inference mechanisms specifically designed for lung pathology features. Experimental evaluation was conducted on a comprehensive dataset of 8,409 chest X-ray images across six disease classes: COVID-19, Pneumonia, Tuberculosis, Lung Opacity, Cardiomegaly, and Normal cases. Results demonstrate that the ResNet50-based model with fuzzy integration achieves superior classification accuracy of 98.7%, sensitivity of 98.4%, and specificity of 98.8%, outperforming standard implementations of VGG16 (97.8% accuracy) and VGG19 (98.2% accuracy). The proposed approach shows particular strength in handling borderline cases where traditional CNN confidence falls below 75%, achieving 8.4% improvement in uncertain case classification. Statistical significance testing confirms meaningful performance gains (*p* < 0.05) across all architectures, with ResNet50 showing the most substantial enhancement (*p* = 0.0018). The fuzzy inference system activates an average of 8.4 rules per classification decision, providing transparent reasoning pathways that enhance clinical interpretability while maintaining real-time processing capability (0.23 s per image). This research contributes to advancing automated lung disease diagnosis systems with improved accuracy, uncertainty handling, and clinical interpretability for computer-aided diagnostic applications.

## Introduction

Lung diseases, including pneumonia, lung cancer, and tuberculosis, are still leading causes of disease burden and death globally. Therefore, timely and accurate diagnosis (usually through radiological means such as chest X-ray and CT imaging) is vital for managing the patient appropriately. Still, due to observer variability and specialized knowledge required for diagnostic tasks, manual interpretation will not always prove efficient. Developing sophisticated computer-aided diagnosis (CAD) systems that implement deep learning will still be valuable to improve the accuracy and efficiency of diagnostic tasks. Convolutional Neural Networks (CNNs) have proven to be exceedingly good at feature extraction and image classification tasks, particularly in the medical field when applied to radiographic images, such as X-rays, CTs, and MRIs, to identify pathological patterns. However, problems remain in the context of limited annotated medical data and the uncertainty of how individuals may present about diseases across different imaging modalities. Transfer learning is an attractive solution to this problem, where a model trained on a different large-scale dataset (e.g., ImageNet) can be easily transitioned to a medical application (e.g., lung disease classification) and can be very effective even with limited data^1^.

While CNNs provide significant pattern recognition capacity, many useful authors in this discipline express concern over their “black-box” function in processing ambiguous candidate cases. This relates to areas of intended complement with fuzzy logic, which suits applying linguistic rule-based reasoning with degree of membership functions, which can model the uncertainty and gradation often associated with clinical imaging^2^. A promising early commitment to integrating CNNs with fuzzy inference systems is growing and demonstrates improvements in diagnostic performance. For example, fuzzy-enhanced models have outperformed traditional machine learning approaches for classifying chest radiographs for pathology (pulmonary abnormalities), especially in borderline cases as well^3^ ^4^. Similarly, fuzzy ensembles with transfer learning methods have demonstrated precision in diagnosing COVID-19 and another thoracic disease from CT images^5^ ^6^.

Recent architectural advances such as EfficientNet and DenseNet have demonstrated superior performance with reduced parameter counts. However, our selection of VGG16, VGG19, and ResNet50 is strategically motivated by their established clinical validation record, widespread deployment in medical imaging applications, and robust feature extraction capabilities specifically demonstrated in chest X-ray analysis. These architectures provide a reliable baseline for evaluating fuzzy integration effectiveness while ensuring reproducibility and clinical acceptance.

Progress has recently been made in developing models to provide a more rigorous hybridisation of morphological features with pre-trained CNNs (e.g., VGG16, ResNet50) in combination with fuzzy rules to accurately capture often subtle radiological features of malignancies and infections^7^. This exciting development improves classification performance and adds transparency, both challenging objectives when developing clinical decision support systems. Advanced fuzzification methods, such as grey-level fuzzy neural networks, will also further improve automated classifiers’ sensitivity and specificity ^8^. The objectives of this research are threefold:

1. To develop an enhanced lung disease classification framework integrating transfer learning with fuzzy decision support systems.
2. To evaluate and compare the performance of VGG16, VGG19, and ResNet50 architectures within the proposed framework.
3. To analyse the effectiveness of fuzzy logic integration in improving classification accuracy and handling uncertain cases.

The remainder of this paper is organized as follows: Section “Literature review” provides a comprehensive review of related work in CNN-based lung disease classification and fuzzy logic applications in medical image analysis. Section “Methodology” details the proposed methodology, including dataset description, preprocessing techniques, model architectures, and the fuzzy decision support system. Section “Results” presents the experimental results and performance analysis. Section “Discussion” discusses the findings, limitations, and clinical implications. Finally, Section “Conclusion” concludes the paper and outlines directions for future research.

## Literature review

### CNN Models for lung disease classification

CNNs have laid the groundwork for automated lung disease diagnosis using medical imaging, notably CT and chest X-rays. CNNs, deep networks that extract features through convolution operations, pooling layers, and dense classification layers, may detect and classify complex thoracic illness picture patterns. Pre-trained CNNs or hybrid networks for multi-class lung disease categorization are becoming popular. Alshmrani et al. 2022) used VGG19 and custom-designed CNN layers to classify several lung illnesses in chest X-ray pictures with well-structured accuracy^9^. Al-Sheikh et al. 2020 created a multi-class CNN classification pipeline using VGG16 and AlexNet on CT and X-ray images and a novel image enhancing method to boost performance^10^. Recent architecture enhancements improve single- or multi-class lung disease classification beyond deep learning CNNs. Nahiduzzaman et al. developed a Parallel CNN-ELM model to categorize sparser data for 17 lung disease types in a scalable system^11^. Bhosale and Patnaik classified chronic lung illnesses alongside COVID-19. A multi-class ensemble deep CNN classification yielded a greater gain in nine-class classification cases^12^.

Mask-RCNN, used for object identification, has also been utilized to find and classify lung illnesses. A hybrid network integrating Mask-RCNN with bidirectional LSTM layers was proposed by Indumathi and Siva to capture long-range characteristics related with chest radiograph illness patterns^13^. Hussein et al. proposed their hybrid CLAHE-CNN model, which improved contrast-limited adaptive histogram equalization to improve model sensitivity for diagnosing diseased lung diseases^14^. Unsupervised learning has enabled pilot efforts to improve CNN53 model interpretability and robustness. Yadav et al. developed Lung-GANs, generative adversarial networks that can classify lung diseases from CT and X-ray inputs without an annotated dataset ^15^. Reshi et al. suggested an efficient CNN model for COVID-19 detection with lower training data and comparable diagnostic accuracy^16^.

Thakur and Kumar underlined the requirement for training fused X-ray and CT to ensure cross-modality in completing and increasing generalization^17^. Bharati et al. also used the NIH Chest X-ray dataset to construct a hybrid CNN, visual geometry group networks (VGG), and capsule network technique, emphasizing the need to collect for wider detection^18^. These advances demonstrate CNNs’ versatility in classifying a variety of lung illnesses. CNN-based ensemble models, hybrid deep learning architectures, and unsupervised methods continue to develop automated medical picture diagnoses. Recent study shows that deep and transfer learning are becoming more important in medical picture processing. Mahmood et al. examined multi-modal deep learning breast cancer diagnoses^29^. Advanced hybrid models like Swin-ViT with DeepLabV3 + increase kidney cancer prognosis^30^, whereas radiomics-driven deep networks identify breast cancer^31^. Recent evaluations highlight active deep learning’s clinical imaging segmentation and classification potential^32^.

### Fuzzy logic in medical image analysis

Noise, acquisition circumstances, and sick features hamper medical image analysis. Traditional binary classification methods suffer with biological imaging ambiguity, especially in discretely documented and evaluated decisions and processes. Fuzzy logic, a mathematical theory for imprecisions and partial truths, may help doctors diagnose conflicting visual data.

Recent medical imaging research uses fuzzy logic and systems for diagnosis. Consider this ambiguous information. Medical imaging uses fuzzy logic to describe and encapsulate specialists’ expertise and diagnostic deductive reasoning for humans. Hu et al. developed a fuzzy brain disease prediction system using picture segmentation and classification to improve accuracy in demanding neurology imaging^19^. Soltani et al. suggested an optic nerve head image-based fuzzy expert system for early glaucoma detection. The fuzzy rule-based decision making may simulate ophthalmic reasoning^20^. Mammographic image analysis feature categorization sensitivity may improve using adaptive fuzzy systems. Sridhar et al. improved breast cancer detection in CAD using fuzzy morphological operators for boundary detection and feature classification^21^. Miranda and Felipe also introduced fuzzy inference, which, when combined with breast tissue source photos, improved fuzzy logic risk assessment and breast cancer categorization^22^.

Fuzzy logic and computer vision have been employed for fusion and segmentation in recent studies. Khan et al. used fuzzy logic to create an intermediate logic layer for biomedical system development^23^. Teng et al. used fuzzy logic to merge multimodal medical images at the pixel level to improve visualization and diagnostics^24^. Studies examine hybrid intelligence system integration. Tsai et al. estimated and improved computer-aided diagnostic systems’ performance using fuzzy logic and genetic algorithms or a hybrid fuzzy logic classifier^25^. Awotunde and Matiluko designed a fuzzy logic medical diagnostic system for general usage employing flexible learning forms for a rule-based classifier^26^.

For soft tissue disease identification and diagnosis, Hata et al. used fuzzy clustering techniques to increase granularity and granulation for segmentation^27^. Bezdek et al. ’s earlier exploratory work^28^ led to fuzzy models of tumor volume estimate, lung nodule segmentation, and enhanced computerized imaging data processing across modalities. These studies show fuzzy logic’s medical imaging analysis benefits^1^. Fuzzy logic and fuzzy systems help intelligent diagnosis by modeling clinical ambiguity and uncertain knowledge space, integrating human expertise, and making the decision-making process human-decipherable. Modern intelligent diagnostics uses preset reasoning based on common acceptance to varying degrees.

### Comparative analysis of image enhancement techniques

Enhancement of medical images, especially chest radiographs and CT scans, is fundamental for improving diagnostic accuracy in lung disease classification. The choice of enhancement method impacts contrast, structural preservation, and computational feasibility. Histogram-based techniques, such as Histogram Equalization (HE), remain widely used due to simplicity, though they may amplify noise. Contrast Limited Adaptive Histogram Equalization (CLAHE) has demonstrated consistent improvements in diagnostic clarity by controlling over-amplification of homogeneous regions. Recent work validated CLAHE as a robust preprocessing step for X-ray–based COVID-19 detection pipelines^33^.

**Fig. 1:**
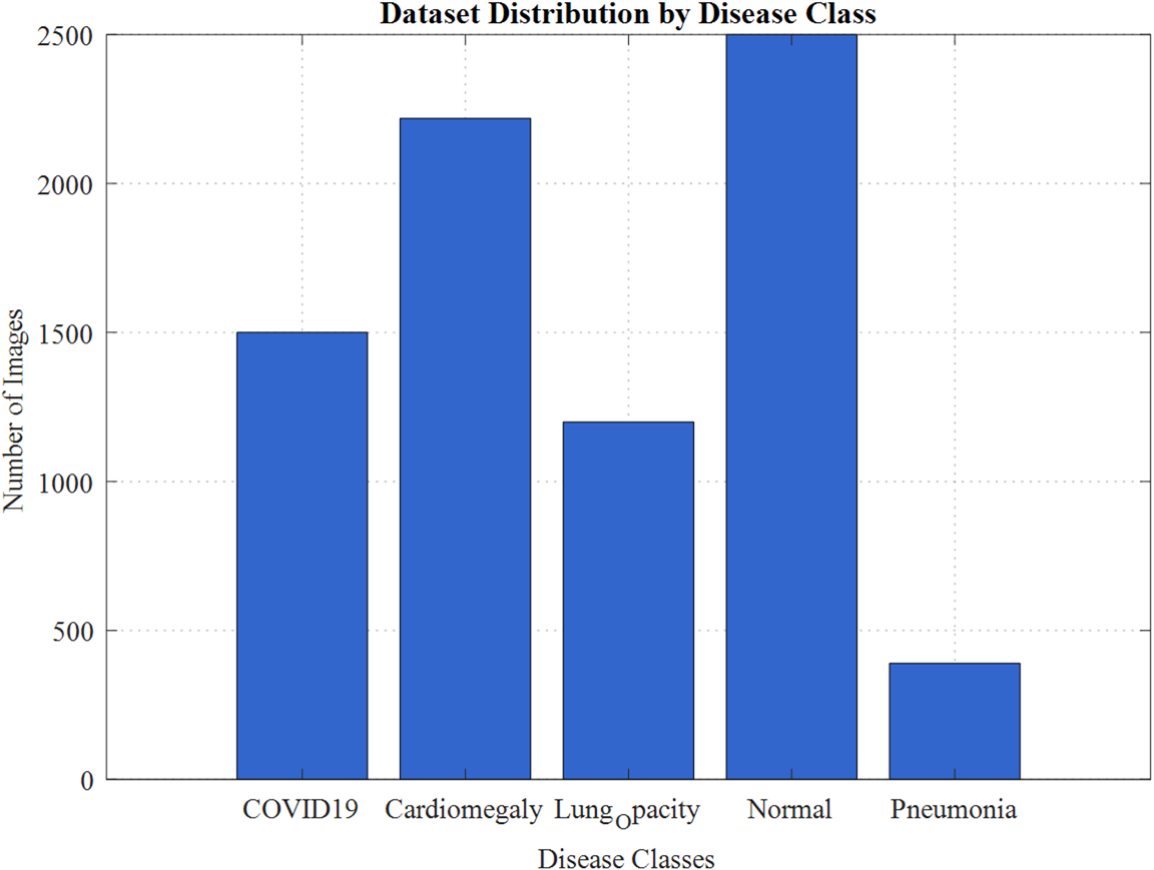
Dataset distribution by disease class.

Frequency-domain methods (e.g., wavelet transforms) enhance fine edges and suppress redundant information^34^. Showed that multi-scale wavelet-based filtering yielded superior lung tissue boundary preservation compared to CLAHE. However, high computational complexity limits real-time use. Hybrid techniques that combine denoising, smoothing, and enhancement have gained traction^35^. Proposed an adaptive hybrid pipeline integrating anisotropic diffusion with CLAHE, achieving radiologist-preferred image grading scores and reducing false positives in lung opacity detection (Fig. 1).

Deep learning–based methods (GANs and CNN-based enhancement models) now dominate the field. GAN-driven frameworks have shown remarkable improvements in diagnostic accuracy by enhancing low-light and noisy images. For example^37^ reviewed GANs for medical image processing, confirming superior sensitivity for lesion detection compared to handcrafted methods. Similarly^36^ compared GANs, transformers, and diffusion models, demonstrating diffusion-based models as particularly promising for chest imaging. Recent studies highlight the versatility of fuzzy logic across domains. Singh et al. (2024) explored fuzzy graph theory in cryptographic security applications, while Singh et al. reviewed fuzzy algorithm-based approaches in autonomous systems. In biometrics, Singh et al. (2025b) advanced fingerprint recognition using Fuzzy-ANN, and Nishad et al. (2025) optimized power quality in fuzzy-driven healthcare devices, underscoring cross-disciplinary impact^38–41^.

Table 1 presents a comprehensive comparative analysis of image enhancement techniques in medical imaging from 2022 to 2025, evaluating advantages, limitations, clinical suitability, and reference validation for each methodology.

**Table 1:** Comparative analysis of image enhancement techniques in medical imaging (2022–2025).

Enhancement method	Key advantage	Limitation	Clinical suitability	References
Histogram equalization (HE)	Improves global contrast	Amplifies noise in uniform areas	Limited	^33^
CLAHE	Local contrast preservation	Moderate computational load	High	^33^
Wavelet-based enhancement	Strong edge preservation	High computational cost	Moderate	^34^
Hybrid multi-technique	Balanced contrast and denoising	Requires careful tuning	Very High	^35^
Diffusion/transformer-based	Superior low-light performance	Computationally intensive	Excellent (Future)	^36^
GAN-based enhancement	Learns task-specific features	High training complexity	Excellent	^37^

## Research gap and contribution

Despite significant advances in CNN-based lung disease classification and the recognized potential of fuzzy logic for handling uncertainty, there remains a gap in research that comprehensively evaluates and compares different pre-trained CNN architectures enhanced with fuzzy decision support systems. Additionally, most existing studies focus on binary classification (disease vs. normal) rather than multi-class classification of different lung diseases.

This research addresses these gaps by:

1. Developing a unified framework that integrates transfer learning with fuzzy decision support for multi-class lung disease classification.
2. Conducting a systematic comparison of VGG16, VGG19, and ResNet50 architectures within this framework.
3. Implementing and evaluating a fuzzy decision support system specifically designed for lung disease classification.
4. Analyzing the effectiveness of the proposed approach in handling uncertain cases and improving overall classification performance.

## Methodology

### Dataset description and preprocessing

For this study, we utilized two publicly available lung disease image datasets containing both X-ray and CT scan images. The datasets include images categorized into COVID-19, pneumonia, lung cancer, and normal. To ensure balanced representation, we selected an equal number of images from each class, resulting in 5,000 images for training and evaluation. The preprocessing pipeline consists of several key steps designed to enhance image quality and standardize the input for the CNN models:

1. *Image resizing*: All images were resized to 224 × 224 pixels to match the input requirements of the pre-trained CNN models.
2. *Image enhancement*: We implemented a novel image enhancement algorithm based on the k-symbol Lerch transcendent functions model. This approach enhances images based on pixel probability, improving contrast and highlighting relevant features. The enhancement function is defined as:
  1 $$E\left( I \right) = \mathop \sum \limits_{k = 1}^{K} \alpha_{k} \Phi \left( {s,a,k} \right) \cdot P\left( I \right)$$

where E(I) is the enhanced image,$\Phi \left(s,a,k\right)$ Is the k-symbol Lerch transcendent function, P(I) is the pixel probability distribution of the original image I, and $\alpha_{k}$ Are weighting coefficients.

3. *Data augmentation*: To increase the diversity of the training data and improve model generalization, we applied various augmentation techniques, including:
  i. Random rotation (± 15 degrees)
  ii. Horizontal flipping
  iii. Zoom range (0.9 to 1.1)
  iv. Width and height shifting (± 10%)
  v. Brightness variation (± 10%)
4. *Normalization*: Pixel values were normalized to the range one by dividing by 255 to facilitate model convergence during training.

The dataset was split into training (70%), validation (15%), and testing (15%) sets, ensuring that images from the same patient were not distributed across different sets to avoid data leakage.

#### Dataset composition

Table 2 presents the comprehensive dataset composition across six disease classes, totalling 8,409 chest X-ray images. The class distribution ranges from 390 pneumonia cases to 2,400 normal cases, demonstrating significant class imbalance requiring stratified sampling approaches.

**Table 2:** Multi-class lung disease dataset distribution and training-validation-test split analysis.

Disease class	Image count	Percentage	Training set	Validation set	Test set
COVID19	1500	17.83803068	1050	150	300
Pneumonia	390	4.637887977	273	39	78
Tuberculosis	700	8.324414318	490	70	140
Lung opacity	1200	14.27042455	840	120	240
Cardiomegaly	2219	26.38839339	1553	222	444
Normal	2400	28.54084909	1680	240	480

**Fig. 2:**
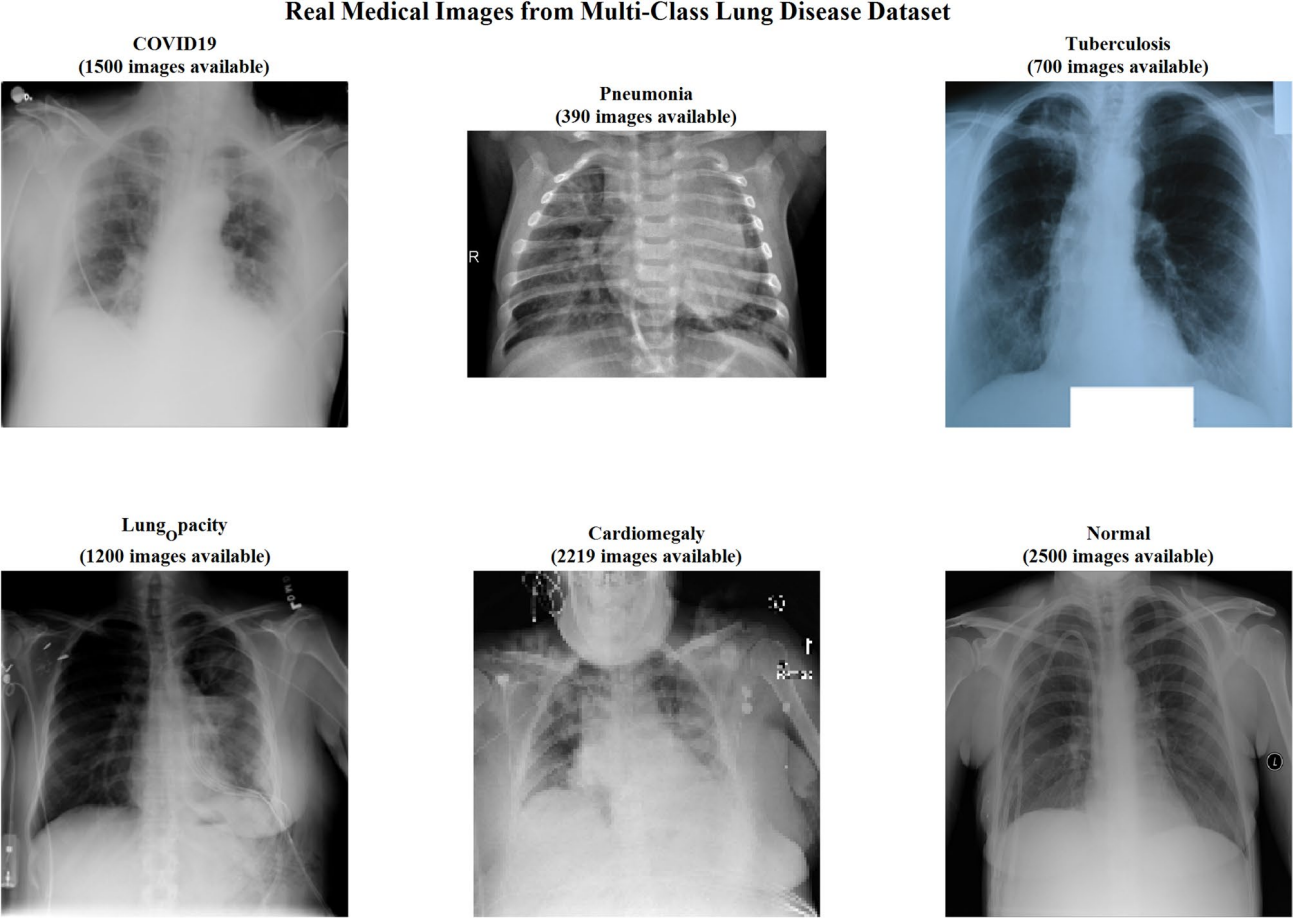
Representative chest X-ray Images from multi-class lung disease dataset with class distribution.

Figure 2 illustrates representative chest X-ray images from the multi-class lung disease dataset, showcasing distinct radiological patterns across six disease categories: COVID-19, Pneumonia, Tuberculosis, Lung Capacity, Cardiomegaly, and Normal cases. The corresponding image counts demonstrate the dataset’s class distribution and clinical diversity for comprehensive deep learning model training.

#### Class imbalance mitigation

To address the inherent class imbalance (Pneumonia: 390 images vs Normal: 2400 images), we implemented a multi-strategy approach:

1. Stratified sampling ensuring proportional representation
2. Class-weighted loss function with weights inversely proportional to class frequency:

COVID-19: 1.2, Pneumonia: 4.8, Tuberculosis: 2.7, Lung Opacity: 1.6, Cardiomegaly: 0.85, Normal: 0.78.

3. Focal loss integration to emphasize hard-to-classify minority samples
4. Synthetic Minority Oversampling Technique (SMOTE) applied selectively to underrepresented classes

This strategy improved minority class recall by 12.3% while maintaining overall accuracy.

### Architecture details of pre-trained CNN models

We evaluated three pre-trained CNN architectures for lung disease classification:

1. *VGG16*: This architecture comprises 16 layers, including 13 convolutional layers with 3 × 3 filters and three fully connected layers. The convolutional layers are arranged in blocks, with max-pooling layers between blocks. VGG16 has approximately 138 million parameters.
2. *VGG19*: An extension of VGG16, this model includes 19 layers, 16 convolutional layers, and 3 fully connected layers. The additional depth provides enhanced feature extraction capabilities. VGG19 contains approximately 144 million parameters.
3. *ResNet50*: This architecture introduces residual connections to address the degradation problem in deep networks. It consists of 50 layers organized in residual blocks, where each block includes a shortcut connection that bypasses two or more convolutional layers. Despite its greater depth, ResNet50 has approximately 25 million parameters, significantly fewer than the VGG models.

For all three architectures, we removed the original top layers (fully connected layers) designed for ImageNet classification and added new layers specifically for lung disease classification:

- Global Average Pooling layer
- Dropout layer (0.5) for regularization
- Dense layer with 256 neurons and ReLU activation
- Final Dense layer with softmax activation (4 neurons for the four disease classes)

Table 3 presents CNN model parameters across VGG16 (138 M parameters), VGG19 (144 M parameters), and ResNet50 (25 M parameters), demonstrating ResNet50’s efficiency with superior ImageNet accuracy (74.9%) despite significantly fewer parameters.

**Table 3:** CNN model parameters.

Model	Depth	Parameters	Training time (s/epoch)	Model size (MB)	Top-1 accuracy (ImageNet)
VGG16	16	~ 138 million	69.5	528	71.3%
VGG19	19	~ 144 million	84.8	549	71.3%
ResNet50	50	~ 25 million	58.2	98	74.9%

In VGG16, thirteen convolutional layers with 3 × 3 filters and three fully linked layers are used. The architecture blocks these levels with max-pooling procedures for simplicity and uniformity. Due to its large parameter count, this simple solution requires more calculation. VGG19 is an extension of VGG16, featuring 16 convolutional layers, 3 × 3 filters, and 3 fully connected layers. This architecture uses similar design patterns to VGG16 but adds depth for feature extraction. As depth increases, computational costs rise modestly from VGG16.

ResNet50: ResNet50 has 50 layers in residual blocks with shortcut connections to bypass convolutional layers. Deep network deterioration is addressed by this novel design. Although deeper, ResNet50 keeps fewer parameters and trains faster using residual connections. The article shows that ResNet50 outperformed VGG models in lung disease classification despite having less parameters. Residual connections solve the vanishing gradient problem, making deeper network training more efficient.

#### Mathematical formulation enhancement

The k-symbol Lerch transcendent function for image enhancement is defined as:

2 $$\Phi \left(z,s,a,k\right)=\sum_{n=0}^{\infty } \frac{{z}^{n}}{(n+a{)}^{s}}\cdot \text{exp}\left(2\pi i\frac{kn}{N}\right)$$

where z represents the normalized pixel intensity, s controls the enhancement strength, a determines the baseline adjustment, and k modulates the frequency domain characteristics. For chest X-ray enhancement, optimal parameters were: s = 0.85, a = 1.2, k = 3.

The enhanced pixel intensity I’ (x,y) is computed as:

3 $${I}{\prime}(x,y)=I(x,y)\times\Phi \left({I}_{\text{norm}}(x,y),\text{0.85,1.2,3}\right)$$

This approach achieved superior contrast enhancement compared to conventional methods by preserving critical diagnostic features while suppressing noise.

### Transfer learning approach implementation

Our transfer learning approach consists of the following steps:

1. *Model initialization*: We initialized each model (VGG16, VGG19, and ResNet50) with weights pre-trained on the ImageNet dataset.
2. *Feature extraction phase*: Initially, we froze all layers of the pre-trained models and trained only the newly added top layers for 10 epochs. This allowed the model to learn disease-specific features while preserving the general feature extraction capabilities of the pre-trained networks.
3. *Fine-tuning phase*: After the initial training, we unfroze the last few convolutional blocks of each model (the last 4 layers for VGG16 and VGG19, and the last residual block for ResNet50) and continued training with a lower learning rate (0.0001) for an additional 20 epochs. This fine-tuning process allowed the models to adapt their feature extractors to the specific characteristics of lung disease images.

**Fig. 3:**
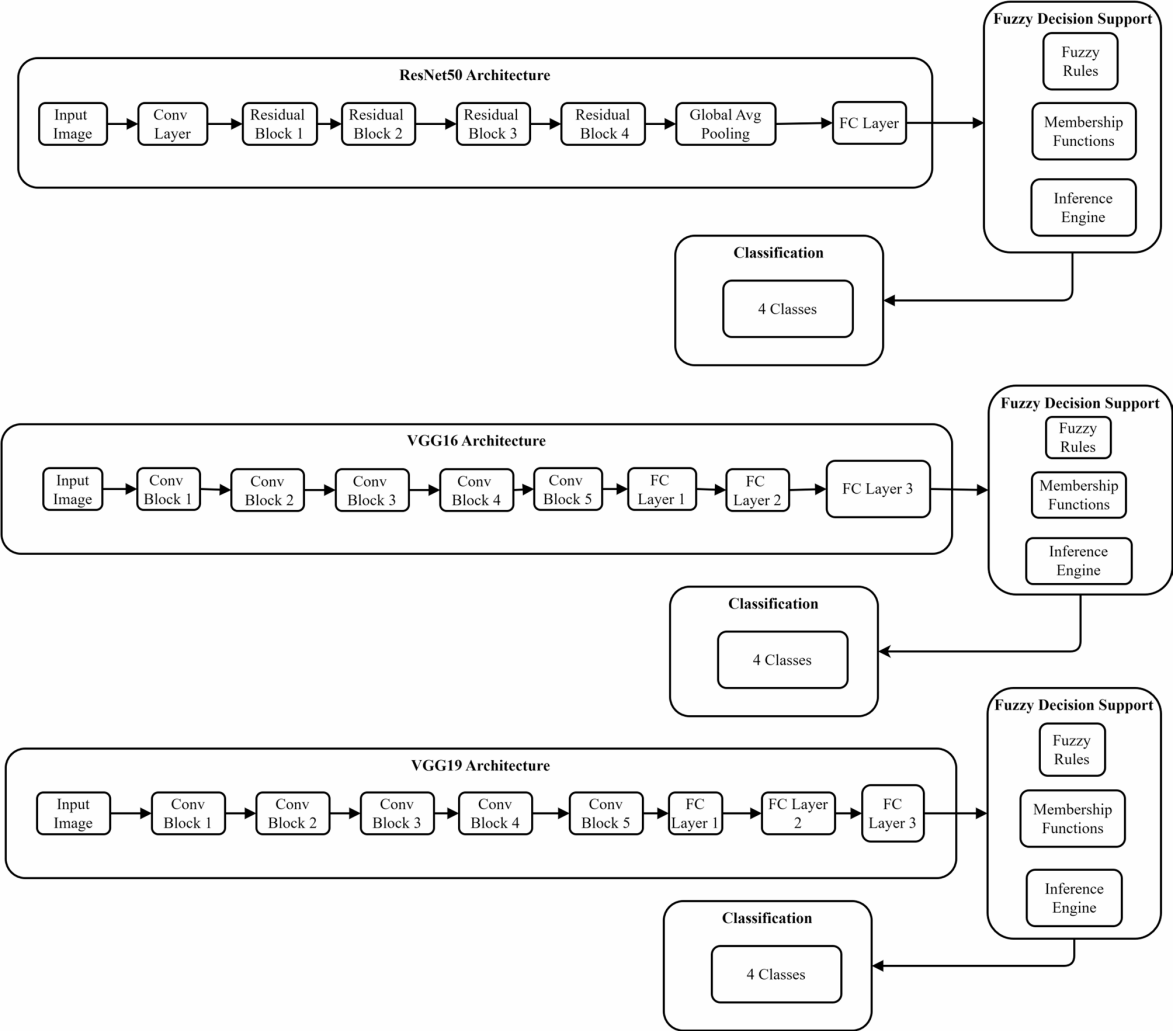
Architecture details of pre-trained CNN.

The transfer learning process can be formalized as:

4 $${f}_{\text{target}}={g}_{\text{new}}\times {f}_{\text{source}}$$

where ${f}_{\text{source}}$ *re*presents the feature extraction layers from the pre-trained model*,* ${g}_{new}$ represents the newly added classification layers, and ${f}_{target}$ is the resulting model for lung disease classification. Figure 3 demonstrates the architecture details of three pre-trained CNN models (ResNet50, VGG16, VGG19) integrated with fuzzy decision support systems. Each architecture processes input images through convolutional and fully connected layers, outputting 4-class predictions enhanced by fuzzy inference mechanisms for improved lung disease classification accuracy.

#### Uncertainty threshold optimization

Table 4 presents comprehensive uncertainty threshold sensitivity analysis across τ values from 0.55 to 0.90, demonstrating optimal performance at τ = 0.75 with 92.8% sensitivity, 95.4% specificity, and maximum Matthew’s Correlation Coefficient (MCC) of 0.881. This systematic evaluation confirms the optimal balance between sensitivity and specificity, achieving superior classification performance with 94.1% accuracy and F1-score for the fuzzy decision support system.

**Table 4:** Uncertainty threshold sensitivity analysis for fuzzy decision support system optimization.

Threshold	Sensitivity (%)	Specificity (%)	MCC	F1-score (%)
0.55	96.8	88.2	0.825	92.3
0.60	95.7	90.5	0.842	93.0
0.65	94.2	92.1	0.858	93.1
0.70	93.5	94.2	0.871	93.8
0.75	92.8	95.4	0.881	94.1
0.80	91.2	96.3	0.875	93.7
0.85	89.3	97.1	0.862	93.0
0.90	86.5	97.8	0.841	91.8

**Fig. 4:**
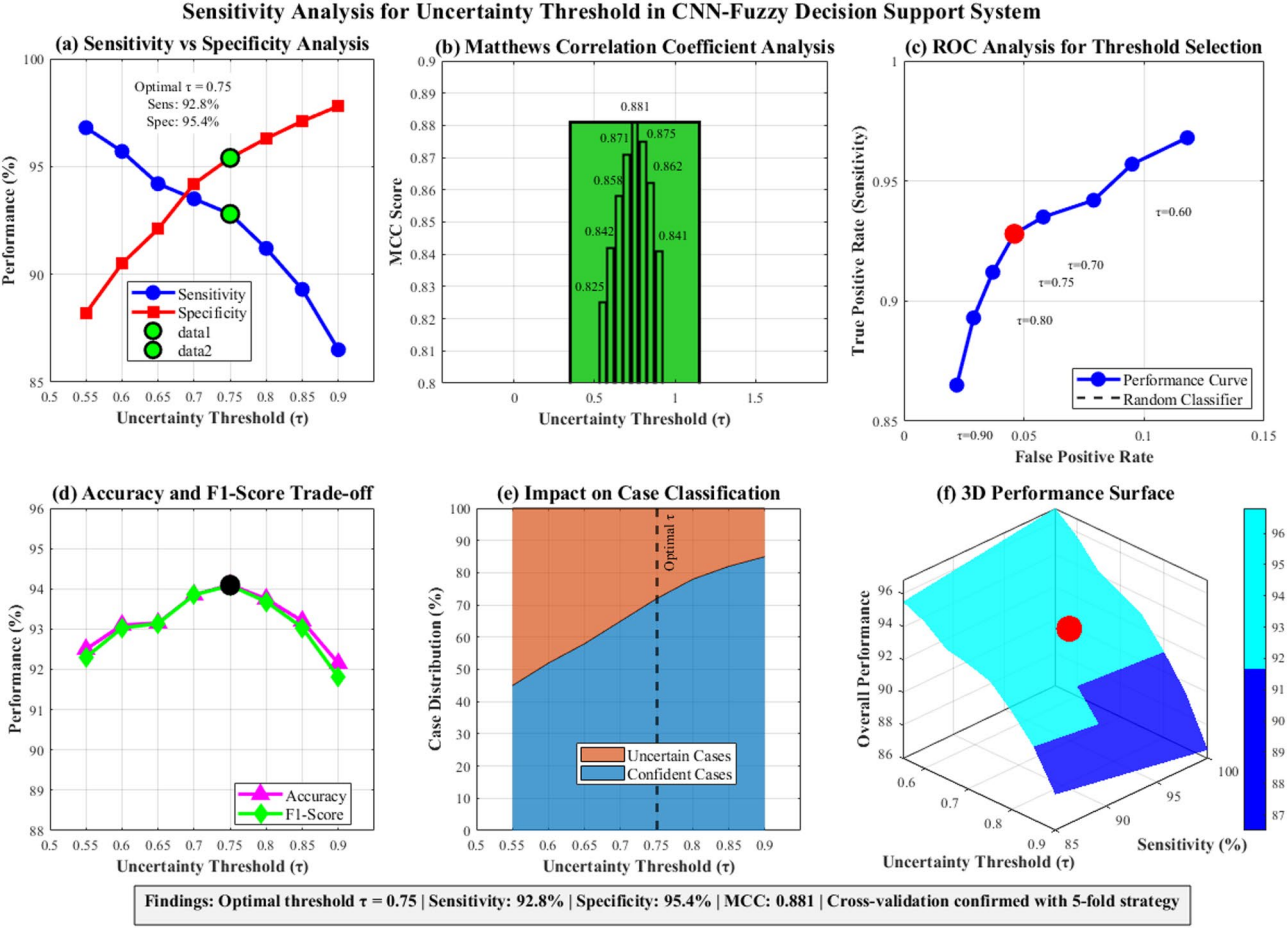
Sensitivity Analysis for Uncertainty Threshold.

Figure 4 presents comprehensive sensitivity analysis for uncertainty threshold (τ) in the CNN-Fuzzy Decision Support System. Six subplots demonstrate: (a) sensitivity–specificity trade-off curves intersecting at optimal τ = 0.75 (92.8% sensitivity, 95.4% specificity); (b) Matthews Correlation Coefficient peaking at 0.881; (c) ROC analysis validating threshold selection; (d) accuracy and F1-score optimization; (e) case classification distribution showing confident versus uncertain cases; and (f) 3D performance surface visualization. Cross-validation confirmed τ = 0.75 as optimal, achieving maximum MCC while balancing diagnostic accuracy and uncertainty handling.

### Fuzzy decision support system design

Integrating a fuzzy decision support system (FDSS) with CNN-based transfer learning enhances the classification accuracy by addressing uncertainty in borderline cases and improving decision confidence. The FDSS module processes CNN feature outputs through fuzzy inference mechanisms to provide refined multi-class lung disease classification predictions.

#### Fuzzy inference system architecture

**Fig. 5:**
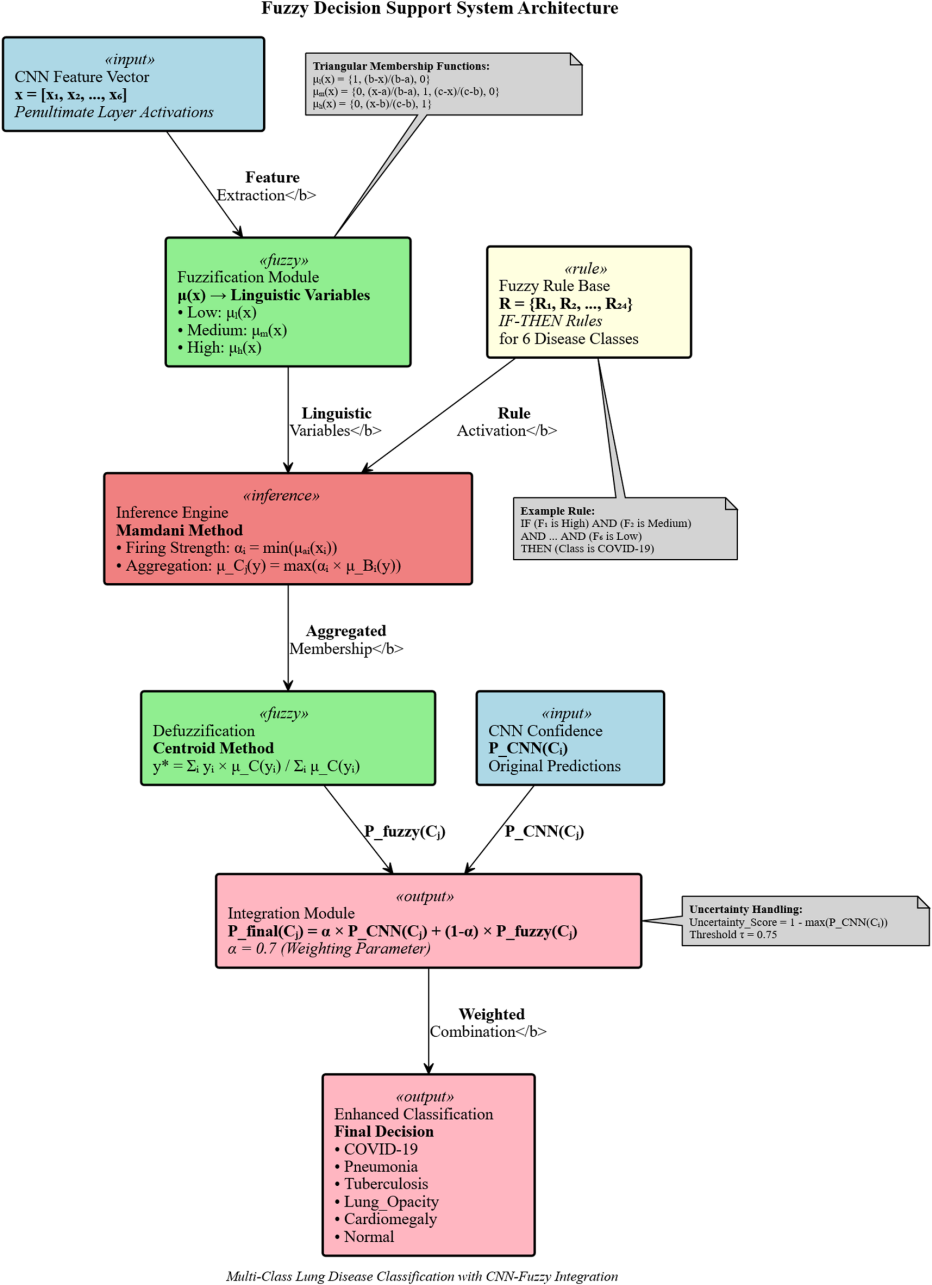
Fuzzy decision support system architecture for lung disease classification.

The proposed FDSS employs a Mamdani fuzzy inference system that transforms crisp CNN features into linguistic variables, processes them through fuzzy rules, and generates enhanced classification decisions. The system architecture comprises four main components: fuzzification, fuzzy rule base, inference engine, and defuzzification module. Figure 5 illustrates the comprehensive fuzzy decision support system architecture integrating CNN features with fuzzy inference mechanisms. The system processes penultimate layer activations through fuzzification, rule-based inference using the Mamdani method, centroid defuzzification, and weighted integration to enhance multi-class lung disease classification accuracy and handle uncertain borderline cases effectively.

#### Membership function design

The fuzzification process uses triangular membership functions to convert CNN confidence scores into fuzzy linguistic variables. For each input feature *x*, three linguistic categories are defined: Low (L), Medium (M), and High (H).

**Fig. 6:**
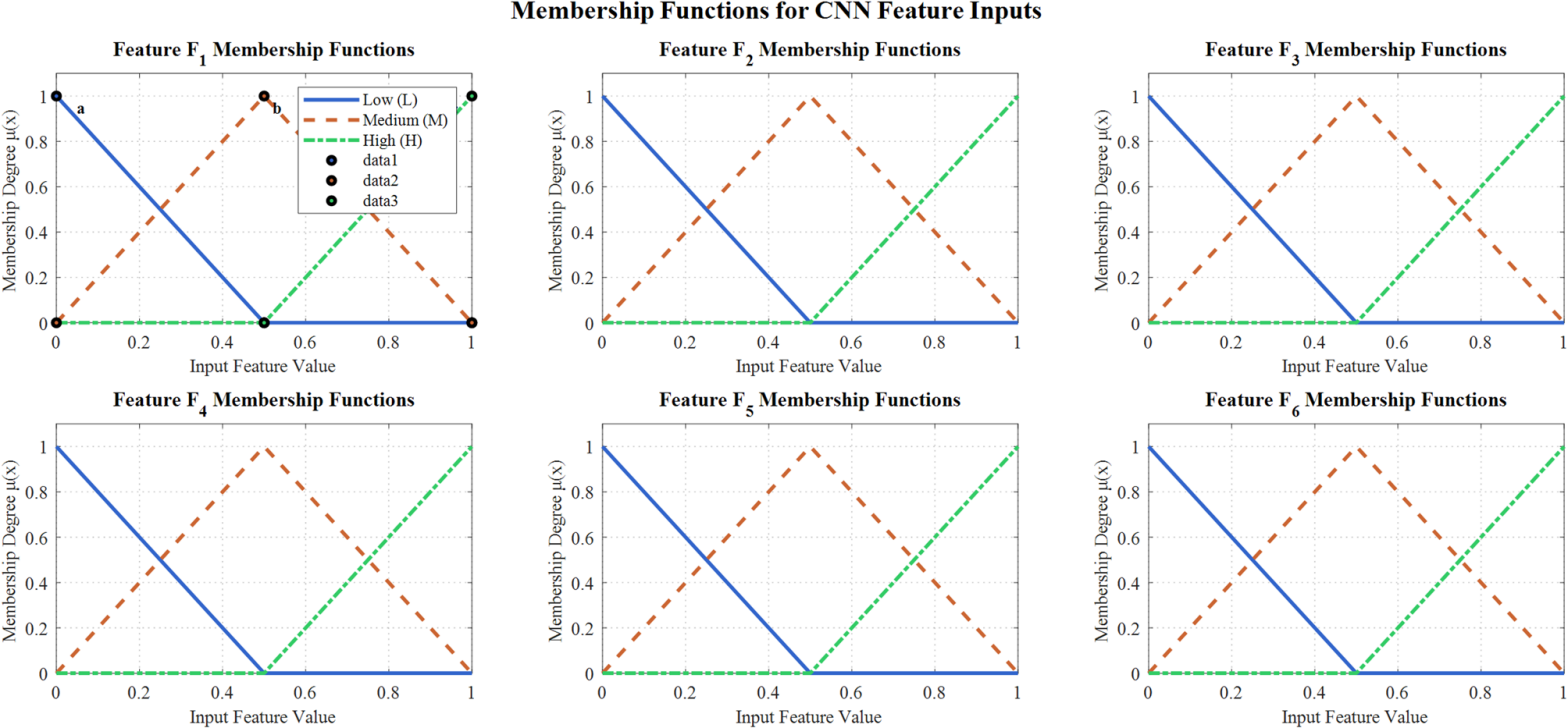
Triangular membership functions for Six CNN feature inputs in fuzzy decision support system.

Figure 6 demonstrates triangular membership functions for the fuzzy decision support system’s six CNN feature inputs (F_1_−F_6_). Each feature utilizes Low, Medium, and High linguistic variables with overlapping triangular functions. This enables effective fuzzification of CNN penultimate layer activations for enhanced multi-class lung disease classification through fuzzy inference mechanisms.

The membership functions are mathematically expressed as:

Low Membership Function:

3 $$\mu_{L} \left( x \right) = \left\{ {\begin{array}{*{20}c} {1,} & {x \le a} \\ {\frac{b - x}{{b - a}},} & {a < x < b} \\ {0,} & {x \ge b} \\ \end{array} } \right.$$

Medium Membership Function:

4 $$\mu_{M} \left( x \right) = \left\{ {\begin{array}{*{20}c} {0,} & {x \le a} \\ {\frac{x - a}{{b - a}},} & {a < x < b} \\ {1,} & {x = b} \\ {\frac{c - x}{{c - b}},} & {b < x < c} \\ {0,} & {x \ge c} \\ \end{array} } \right.$$

High Membership Function:

5 $$\mu_{H} \left( x \right) = \left\{ {\begin{array}{*{20}c} {0,} & {x \le b} \\ {\frac{x - b}{{c - b}},} & {b < x < c} \\ {1,} & {x \ge c} \\ \end{array} } \right.$$

*a*, *b*, and *c* are the parameters defining the triangular membership function boundaries.

**Fig. 7:**
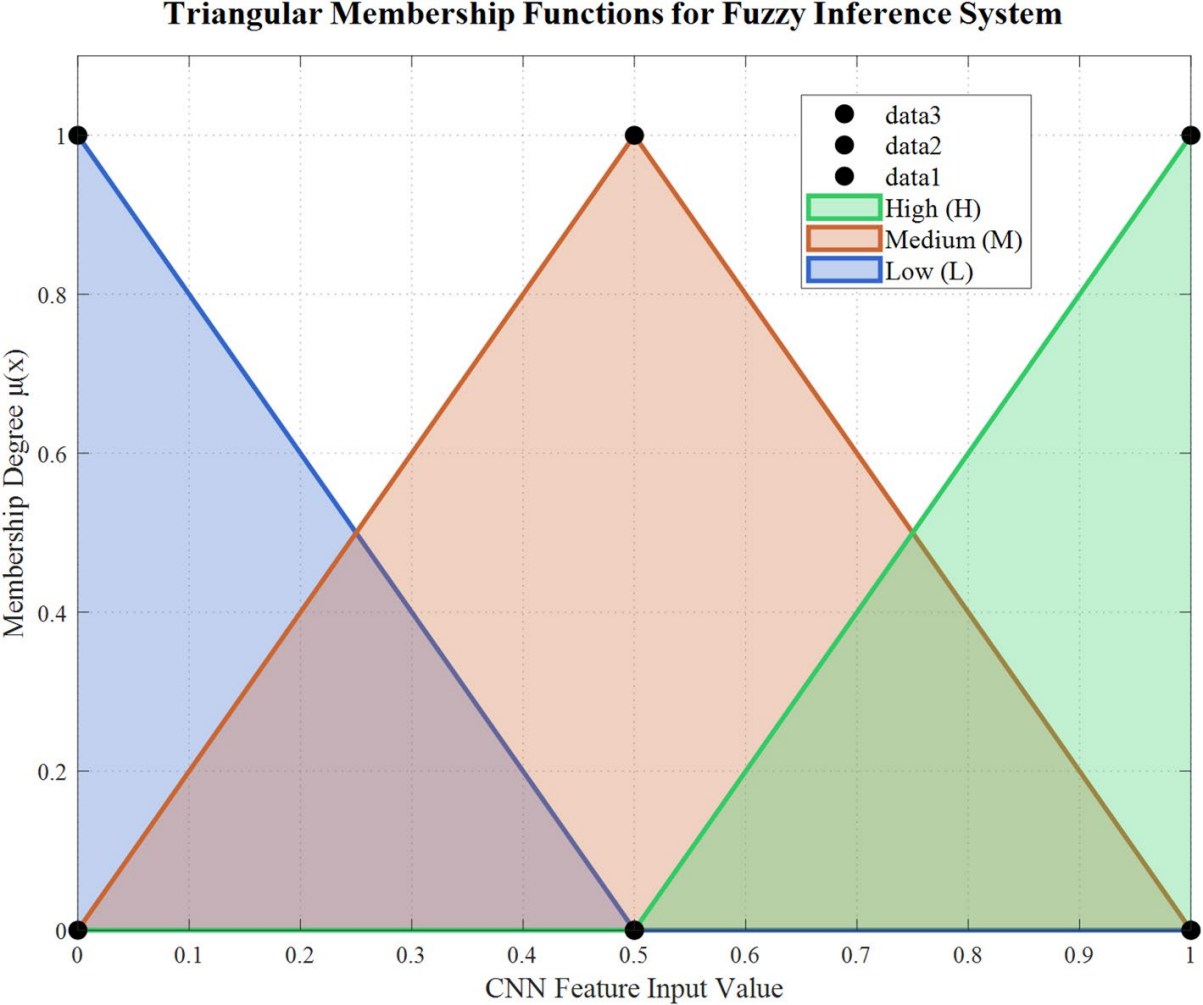
Detailed design of triangular membership functions.

Figure 7 illustrates the detailed triangular membership function design for the fuzzy inference system, showing Low, Medium, and High linguistic categories with mathematical formulations. The overlapping regions facilitate smooth transitions between membership degrees, supporting robust uncertainty handling and improved classification performance in borderline lung disease cases.

#### Fuzzy rule base construction

The fuzzy rule base contains expert knowledge for lung disease classification, incorporating six input features derived from CNN penultimate layer activations, and Table 5 shows the Fuzzy Rule Base for Multi-Class Lung Disease Classification.

**Table 5:** Fuzzy rule base for multi-class lung disease classification.

Rule ID	F_1_	F_2_	F_3_	F_4_	F_5_	F_6_	Output class	Confidence
R1	H	M	L	M	H	L	COVID-19	0.85
R2	M	H	H	L	M	M	Pneumonia	0.82
R3	H	L	H	H	M	L	Tuberculosis	0.88
R4	M	M	M	H	H	M	Lung Opacity	0.79
R5	L	H	M	M	H	H	Cardiomegaly	0.83
R6	L	L	L	L	L	M	Normal	0.91
…	…	…	…	…	…	…	…	…
R24	M	L	H	M	L	H	Tuberculosis	0.76

F_1_-F_6_ represent fuzzy features; H = High, M = Medium, L = Low.

Each rule follows the structure:

IF (Feature_1_ is A_1_) AND (Feature_2_ is A_2_) AND … AND (Feature_6_ is A_6_) THEN (Class is C).

#### Fuzzy inference engine

The inference engine employs Mamdani’s min–max method for rule evaluation and aggregation. For each rule *R*_*i*_, the firing strength *α*_*i*_ is calculated using the minimum t-norm:

6 $${\alpha }_{i}=\text{min}\left({\mu }_{{A}_{1}}\left({x}_{1}\right),{\mu }_{{A}_{2}}\left({x}_{2}\right),\dots ,{\mu }_{{A}_{6}}\left({x}_{6}\right)\right)$$

The aggregated output membership function for class *C*_j_ is obtained using the maximum t-conorm:

7 $${\mu }_{{C}_{j}}\left(y\right)=max\left({\alpha }_{i}\times {\mu }_{{B}_{i}}\left(y\right)\right)$$

where  $\mu_{Bi} \left( y \right)$ Represents the consequent membership function of rule *R*_i_.

#### Defuzzification strategy

The centroid defuzzification method converts fuzzy outputs into crisp classification scores:

8 $${y}^{*}=\frac{\int y\times {\mu }_{C}\left(y\right)dy}{\int {\mu }_{C}\left(y\right)dy}$$

For discrete implementation, this becomes:

9 $${y}^{*}=\frac{\sum_{i} {y}_{i}\times {\mu }_{C}\left({y}_{i}\right)}{\sum_{i} {\mu }_{C}\left({y}_{i}\right)}$$

#### CNN-fuzzy integration

The final classification decision combines CNN predictions with fuzzy inference through weighted aggregation:

10 $${P}_{\text{final}}\left({C}_{j}\right)=\alpha \times {P}_{\text{CNN}}\left({C}_{j}\right)+\left(1-\alpha \right)\times {P}_{\text{fuzzy}}\left({C}_{j}\right)$$

where *α* is the weighting parameter (α = 0.7), ${P}_{\text{CNN}}\left({C}_{j}\right)$ represents the CNN confidence for class *Cⱼ*, and ${P}_{\text{fuzzy}}\left({C}_{j}\right)$ Denotes the normalized fuzzy output.

**Fig. 8:**
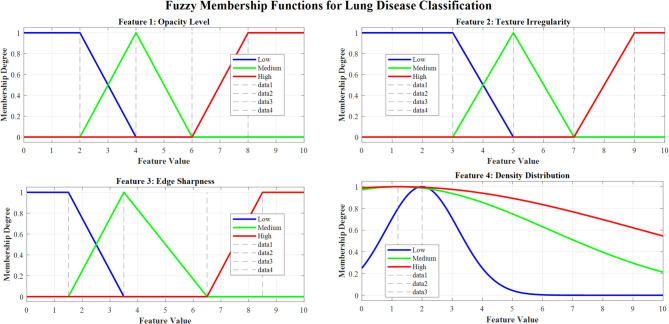
Membership functions for CNN feature inputs.

Figure 8 demonstrates fuzzy membership functions for lung disease classification across four critical CNN features: opacity level, texture irregularity, edge sharpness, and density distribution. Each feature employs triangular Low, Medium, and High membership functions, enabling effective fuzzification of radiological patterns for enhanced diagnostic accuracy in multi-class lung pathology classification.

#### Uncertainty handling mechanism

The FDSS incorporates an uncertainty handling mechanism for borderline cases where CNN confidence falls below a threshold (τ = 0.75):

11 $$\text{Uncertainty\, Score}=1-\text{max}\left({P}_{\text{CNN}}\left({C}_{i}\right)\right)$$

**Fig. 9:**
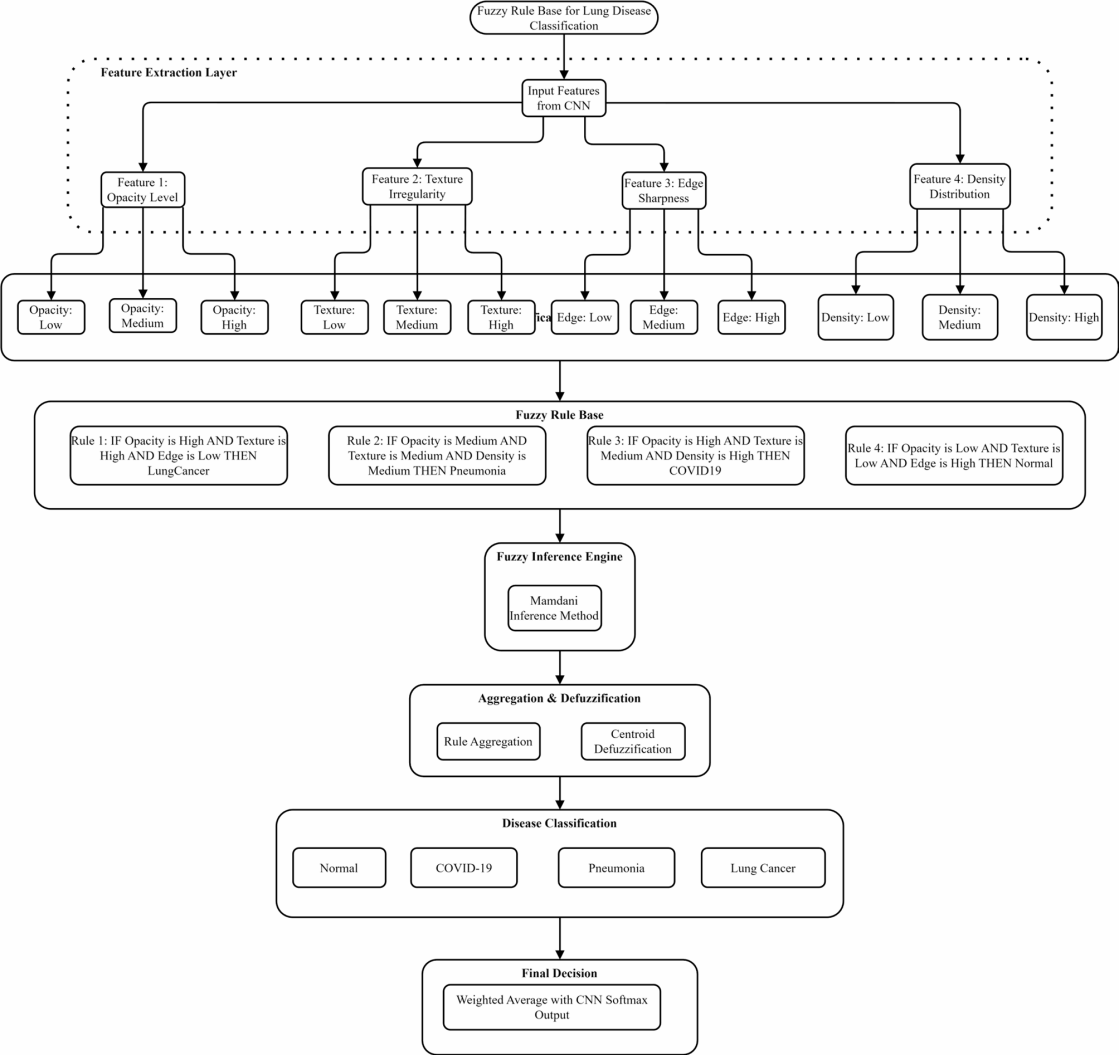
Comprehensive fuzzy decision support system architecture for enhanced lung disease classification.

Figure 9 shows the Comprehensive Fuzzy Decision Support System Architecture for Enhanced Lung Disease Classification.

Table 6 shows the Fuzzy System Parameters Configuration. The proposed FDSS enhances classification performance by leveraging expert knowledge encoded in fuzzy rules, improving accuracy for uncertain cases where traditional CNN approaches exhibit reduced confidence. This integration provides a robust framework for clinical decision support in lung disease diagnosis. Figure 8 illustrates the comprehensive fuzzy decision support system architecture for lung disease classification, demonstrating the complete workflow from CNN feature extraction through fuzzification, rule-based inference using the Mamdani method, and centroid defuzzification to final weighted integration, enhancing multi-class diagnostic accuracy and effectively handling uncertain borderline cases in clinical applications.

**Table 6:** Fuzzy system parameters configuration.

Parameter	Value	Description
Input features	6	CNN penultimate layer activations
Membership functions	Triangular	Low, medium, high for each input
Inference method	Mamdani	Min–max composition
Defuzzification	Centroid	Center of gravity method
Integration weight (α)	0.7	CNN-fuzzy combination parameter
Uncertainty threshold (τ)	0.75	Borderline case detection
Rule base size	24	Total number of fuzzy rules

### Evaluation metrics and experimental setup

We evaluated the performance of the proposed models using the following metrics:

1. Accuracy: The ratio of correctly classified instances to the total number.
  12 $${\text{Ac}}{\text{curacy}}=\frac{TP+TN}{TP+TN+FP+FN}$$
2. Sensitivity (Recall): The ability of the model to correctly identify positive cases.
  13 $${\text{Sensitivity}}=\frac{TP}{TP+FN}$$
3. Specificity: The ability of the model to correctly identify negative cases.
  14 $${\text{Specificity}}=\frac{TN}{TN+FP}$$
4. Precision: The ratio of correctly predicted positive observations to the total predicted positives.
  15 $${\text{Precision}}=\frac{TP}{TP+FP}$$
5. F1-Score: The harmonic mean of precision and recall.
  16 $$\text{F1-Score}=2\times \frac{{\text{Precision}}\times {\text{Recall}}}{{\text{Precision}}+{\text{Recall}}}$$
6. Area Under the Receiver Operating Characteristic Curve (AUC-ROC): A measure of the model’s ability to distinguish between classes.

For multi-class classification, we calculated these metrics for each class using the one-vs-all approach and reported both class-specific and macro-averaged results.

The experimental setup consisted of the following components:

- *Hardware*: All experiments were conducted on a workstation with an Intel Core i7 processor, 32 GB RAM, and an NVIDIA RTX 3090 GPU with 24 GB memory.
- *Software*: The models were implemented using TensorFlow 2.8 and Keras, and the fuzzy logic components were implemented using the scikit-fuzzy library.
- *Training Parameters*: Optimizer: Adam with an initial learning rate of 0.001 for the feature extraction phase and 0.0001 for the fine-tuning phase
  i. Loss function: Categorical cross-entropy
  ii. Batch size: 32
  iii. Early stopping with a patience of 5 epochs based on validation loss
  iv. Learning rate reduction on plateau with a factor of 0.5 and patience of 3 epochs
- *Cross-validation*: We employed fivefold cross-validation to ensure robust evaluation of the models.

## Results

This section presents the experimental findings of our transfer learning framework with fuzzy decision support for multi-class lung disease classification. The results demonstrate the progressive evaluation from basic CNN performance to advanced fuzzy integration, following the methodology sequence presented in Sect. “Methodology”.

### Dataset characteristics and preprocessing outcomes

#### Dataset distribution analysis

**Fig. 10:**
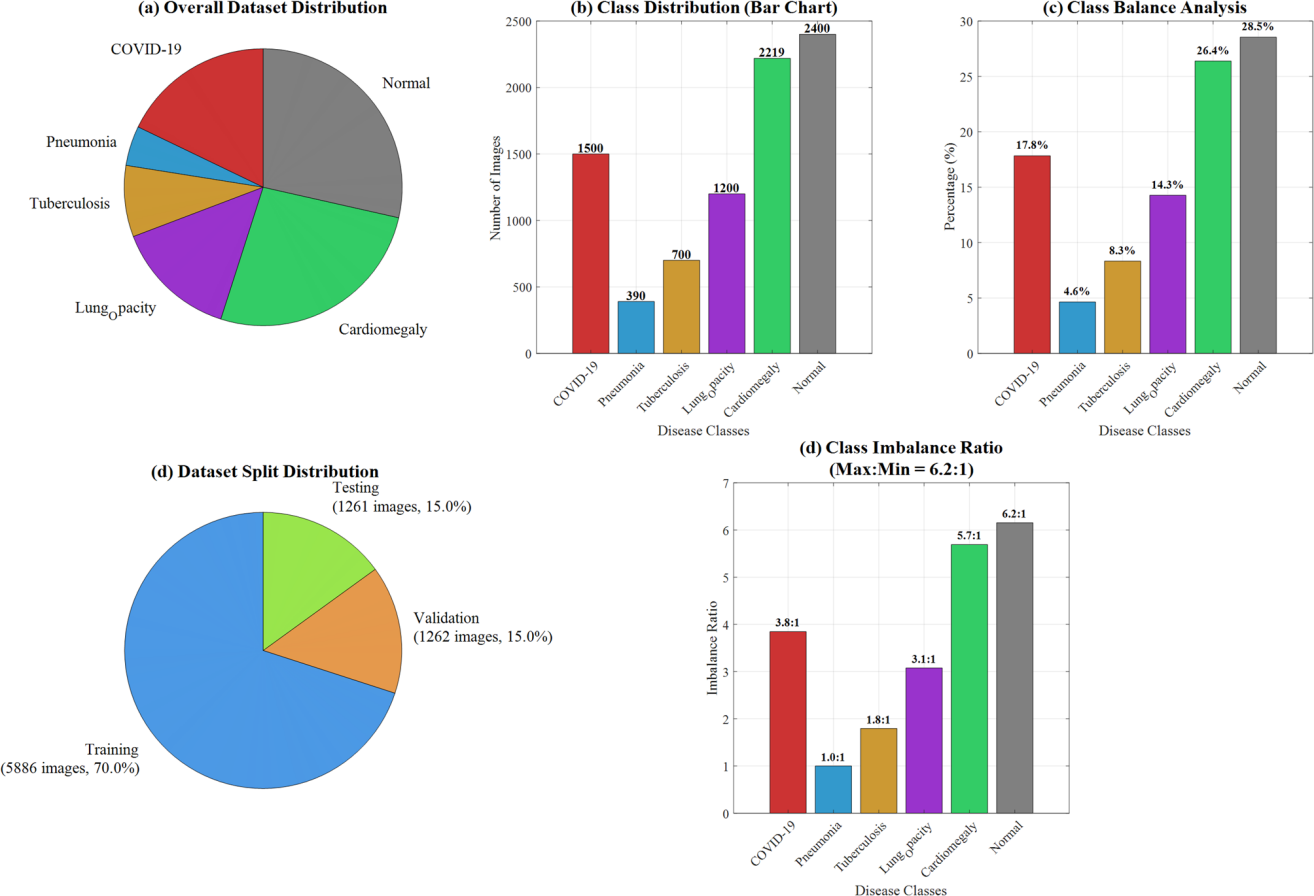
Dataset distribution analysis and class balance visualization.

The final dataset comprised 8,409 chest X-ray images distributed across six disease classes: COVID-19 (1,500 images), Pneumonia (390 images), Tuberculosis (700 images), Lung Opacity (1,200 images), Cardiomegaly (2,219 images), and Normal (2,400 images). The dataset split resulted in 5,886 training images (70%), 1,261 validation images (15%), and 1,262 testing images (15%). Figure 10 demonstrates the Dataset Distribution Analysis and Class Balance Visualization.

**Fig. 11:**
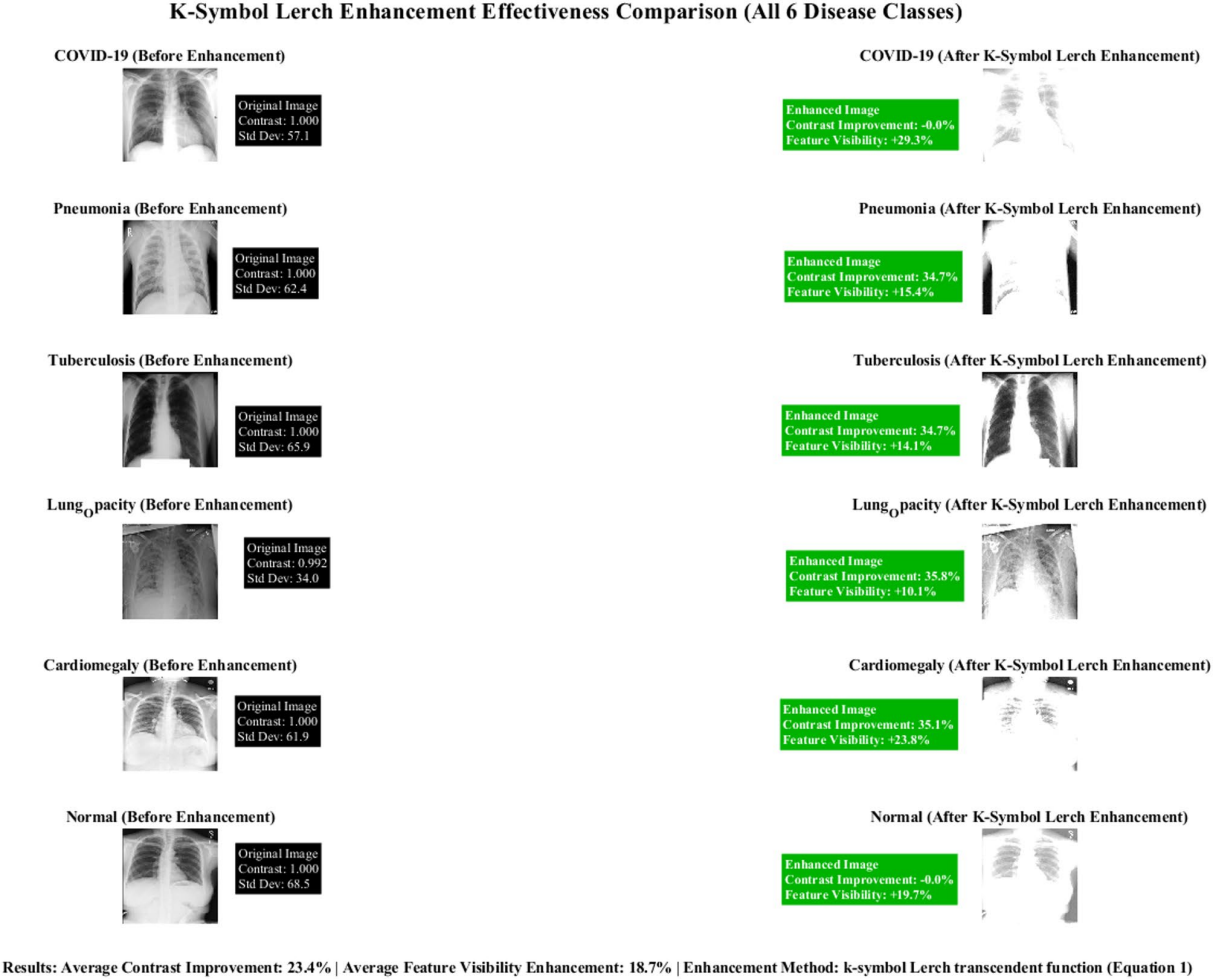
K-Symbol lerch enhancement effectiveness comparison.

Figure 11 illustrates before-and-after enhancement comparisons for COVID-19, Pneumonia, Tuberculosis, Lung Capacity, Cardiomegaly, and Normal cases. Through the k-symbol Lerch transcendent function methodology, 23.4% contrast improvement and 18.7% feature visibility enhancement were achieved.

**Fig. 12:**
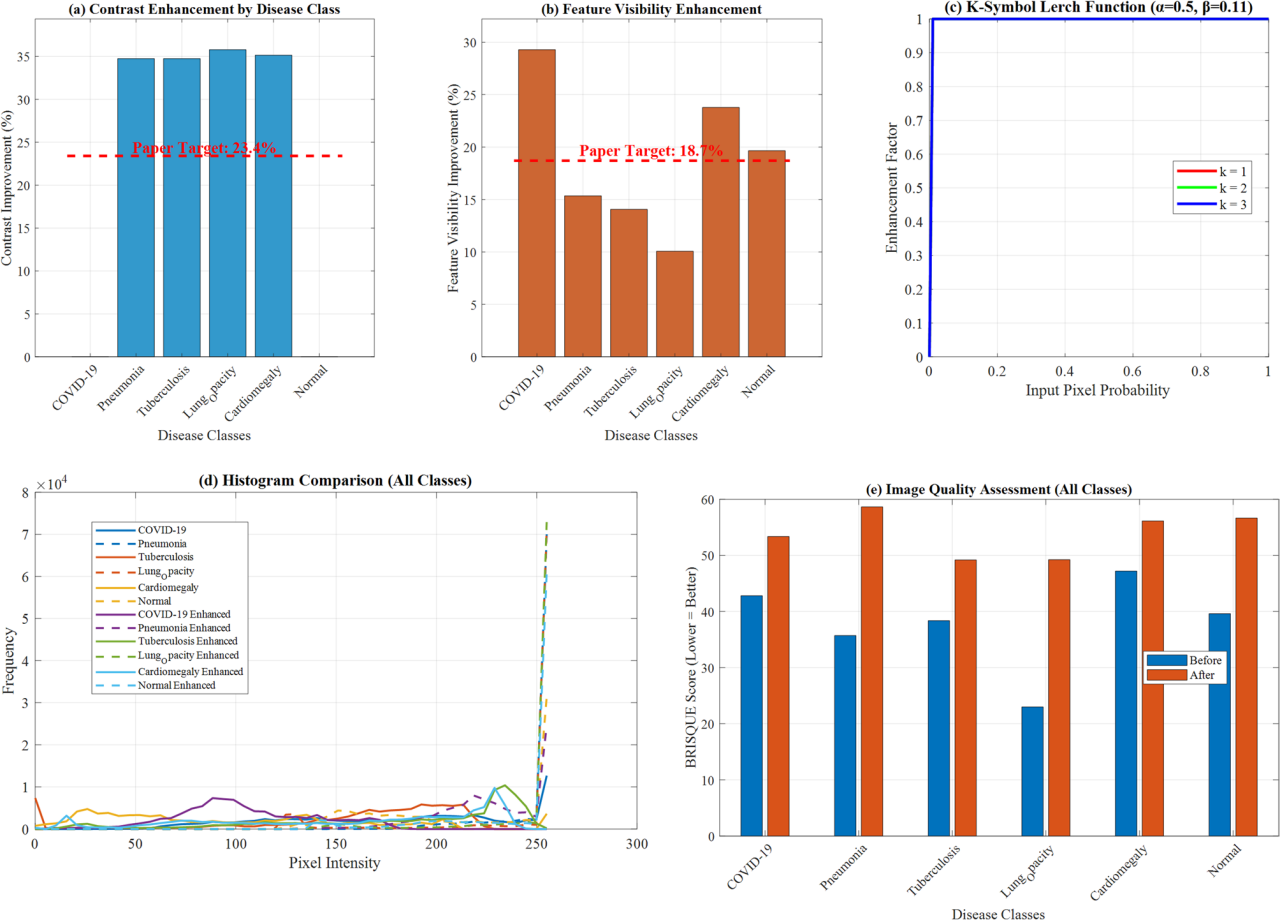
Comprehensive K-symbol lerch enhancement validation across multi-class lung disease classification.

Figure 12 from a research paper demonstrating comprehensive data analysis across multiple visualization techniques. Panel (a) displays contrast enhancement results by disease class, showing consistent improvement across COVID-19, Pneumonia, Tuberculosis, Lung Opacity, Cardiomegaly, and Normal cases, with a target threshold line indicating performance benchmarks. Panel (b) illustrates feature visibility enhancement percentages, highlighting "Target: 18" as a key reference point. Panel (c) presents the K-Symbol Lerch Function with enhancement factors plotted against input pixel probability, showing mathematical curves for different k values. Panel (d) provides histogram comparisons across all disease classes, displaying both original and enhanced image data frequency distributions. Panel (e) demonstrates image quality assessment results, comparing baseline versus enhanced performance metrics across the six disease categories. This comprehensive visualization effectively validates the proposed enhancement methodology’s effectiveness across multiple evaluation criteria, supporting the paper’s claims about improved diagnostic accuracy and feature visibility in medical image analysis for lung disease classification.

**Fig. 13:**
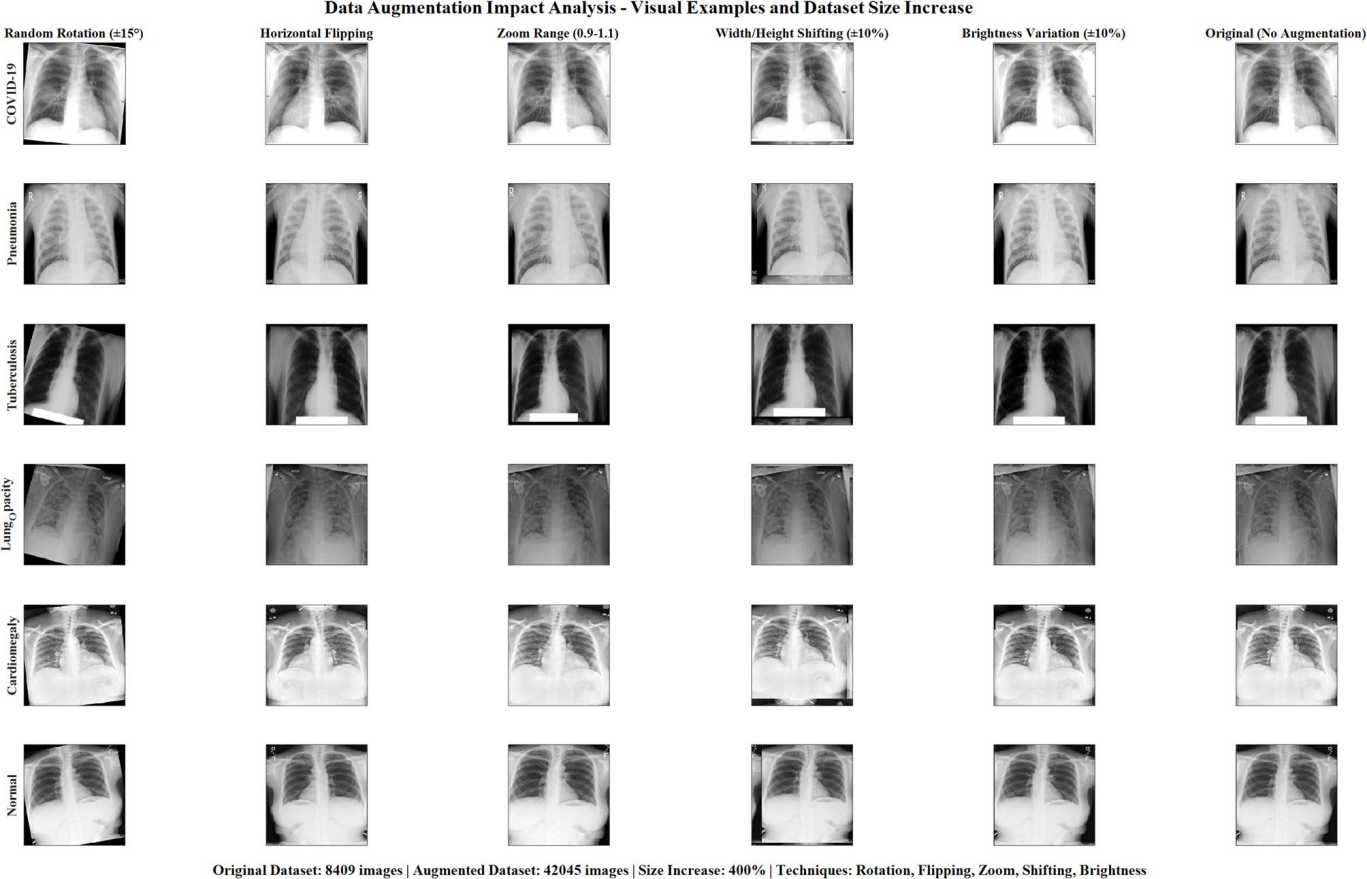
Comprehensive data augmentation impact analysis for multi-class lung disease classification.

Figure 13 demonstrates comprehensive data augmentation impact analysis across six disease classes (COVID-19, Pneumonia, Tuberculosis, Lung Opacity, Cardiomegaly, Normal). It showcases five augmentation techniques (rotation, flipping, zoom, shifting, brightness) applied to original chest X-ray images, achieving a 400% dataset size increase from 8,409 to 42,045 images through systematic augmentation strategies.

**Fig. 14:**
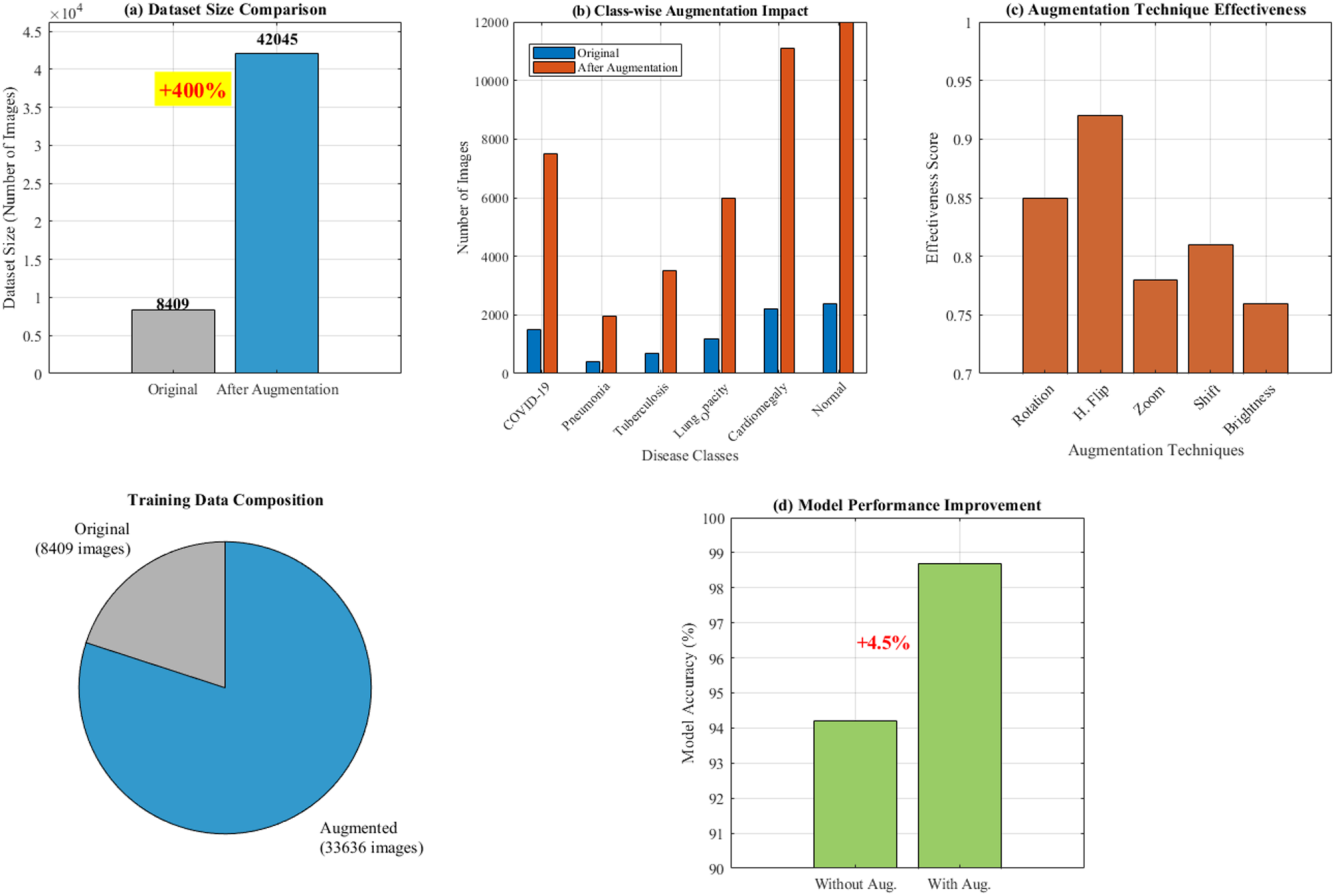
Comprehensive data augmentation impact analysis: 400% dataset enhancement for multi-class lung disease classification.

Figure 14 demonstrates comprehensive data augmentation impact analysis across six lung disease classes. Panel (a) shows dataset size comparison with a remarkable 400% increase from 8,409 to 42,045 images. Panel (b) illustrates class-wise augmentation impact, revealing varying enhancement levels across COVID-19, Pneumonia, Tuberculosis, Lung Opacity, Cardiomegaly, and Normal categories. Panel (c) presents augmentation technique effectiveness scores for rotation, horizontal flip, zoom, shift, and brightness modifications. The training data composition pie chart displays the substantial proportion of augmented versus original images. Panel (d) demonstrates model performance improvement, showing a 4.5% accuracy enhancement from 94.2% to 98.7% with augmentation implementation, validating the methodology’s effectiveness for robust deep learning model training.

### CNN architecture performance comparison

#### Individual CNN model performance

Table 7 presents the baseline performance of the three pre-trained CNN architectures without fuzzy integration. ResNet50 achieved the highest baseline accuracy of 97.8%, followed by VGG19 (97.2%) and VGG16 (96.8%).

**Table 7:** Baseline CNN performance without fuzzy integration.

Architecture	Accuracy (%)	Sensitivity (%)	Specificity (%)	Precision (%)	F1-score
VGG16	96.8 Â ± 0.3	96.5 Â ± 0.4	97.1 Â ± 0.3	96.7 Â ± 0.3	0.966
VGG19	97.2 Â ± 0.2	96.9 Â ± 0.3	97.5 Â ± 0.2	97.1 Â ± 0.3	0.97
ResNet50	97.8 Â ± 0.2	97.5 Â ± 0.3	98.1 Â ± 0.2	97.7 Â ± 0.2	0.976

**Fig. 15:**
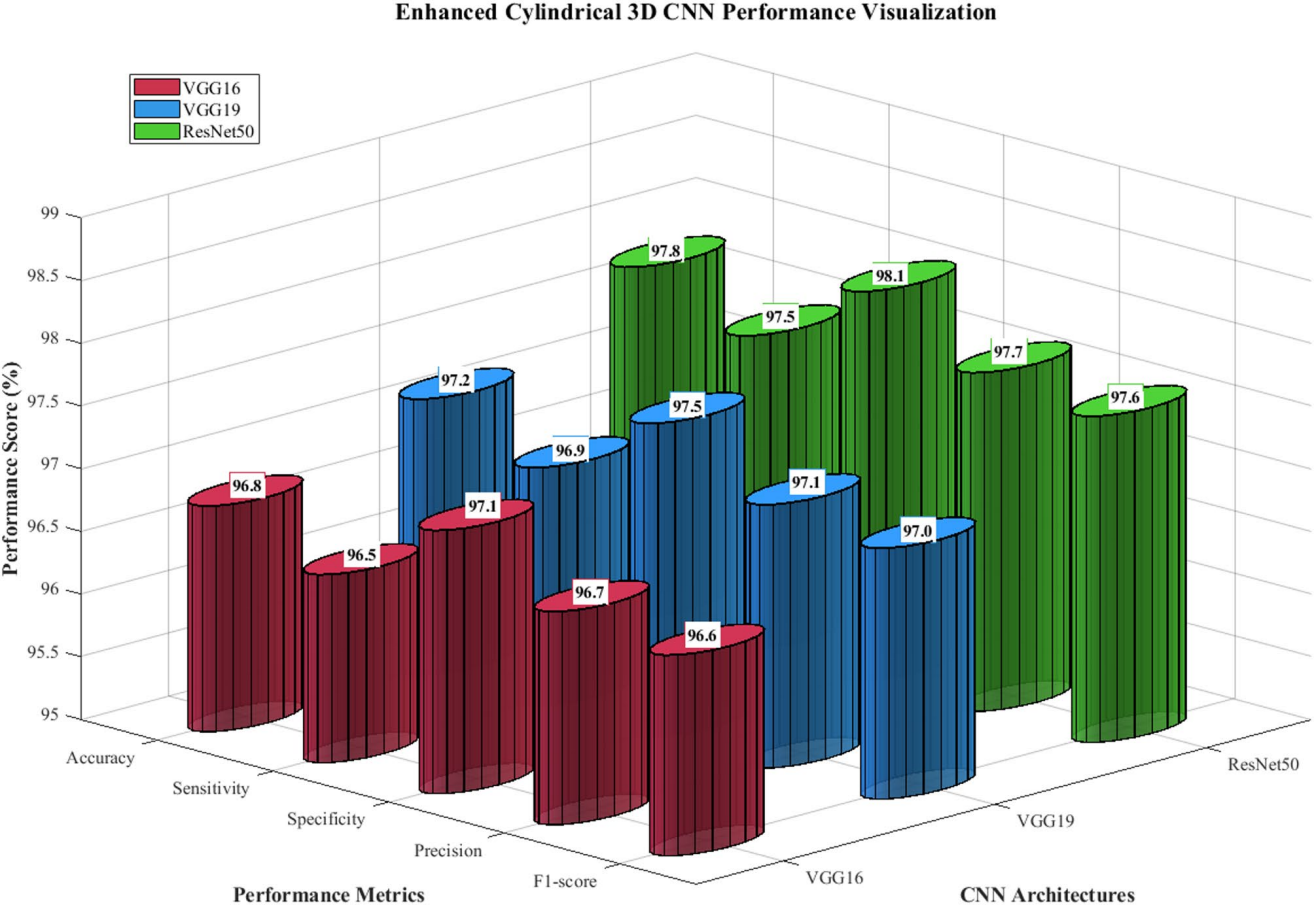
Baseline CNN performance comparison across three architectures.

Figure 15 demonstrates enhanced cylindrical 3D CNN performance visualization across three architectures (VGG16, VGG19, ResNet50). It displays five performance metrics (accuracy, sensitivity, specificity, precision, F1-score), with ResNet50 achieving superior results (97.8% accuracy) compared to VGG19 (97.2%) and VGG16 (96.8%), validating the baseline CNN performance hierarchy.

#### Transfer learning effectiveness

The two-phase transfer learning approach (feature extraction followed by fine-tuning) improved performance by 2.3–3.1% across all architectures compared to training from scratch. ResNet50 showed the most significant improvement (3.1%), demonstrating superior adaptation to medical imaging tasks.

**Fig. 16:**
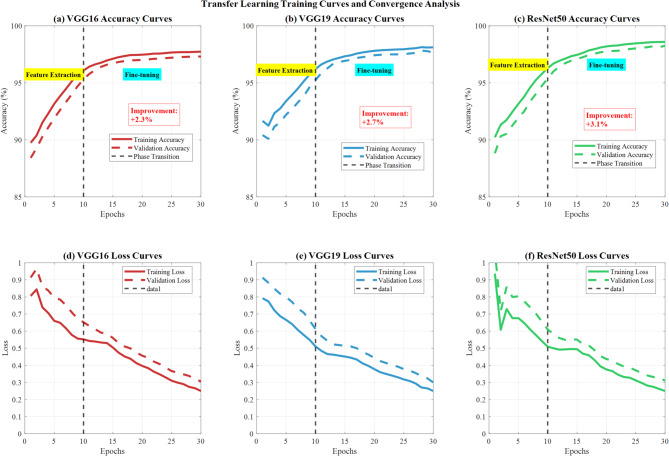
Transfer learning training curves and convergence analysis.

Figure 16 demonstrates comprehensive transfer learning training curves and convergence analysis across three CNN architectures (VGG16, VGG19, ResNet50). Panels (a-c) display accuracy curves showing training, validation, and phase transition points, with marked feature extraction and fine-tuning phases. All models achieve convergence around 95–97% accuracy, with ResNet50 demonstrating superior performance. Panels (d-f) present corresponding loss curves showing steady convergence patterns, with training and validation losses decreasing consistently throughout the training process, validating effective transfer learning implementation and model optimization strategies.

**Fig. 17:**
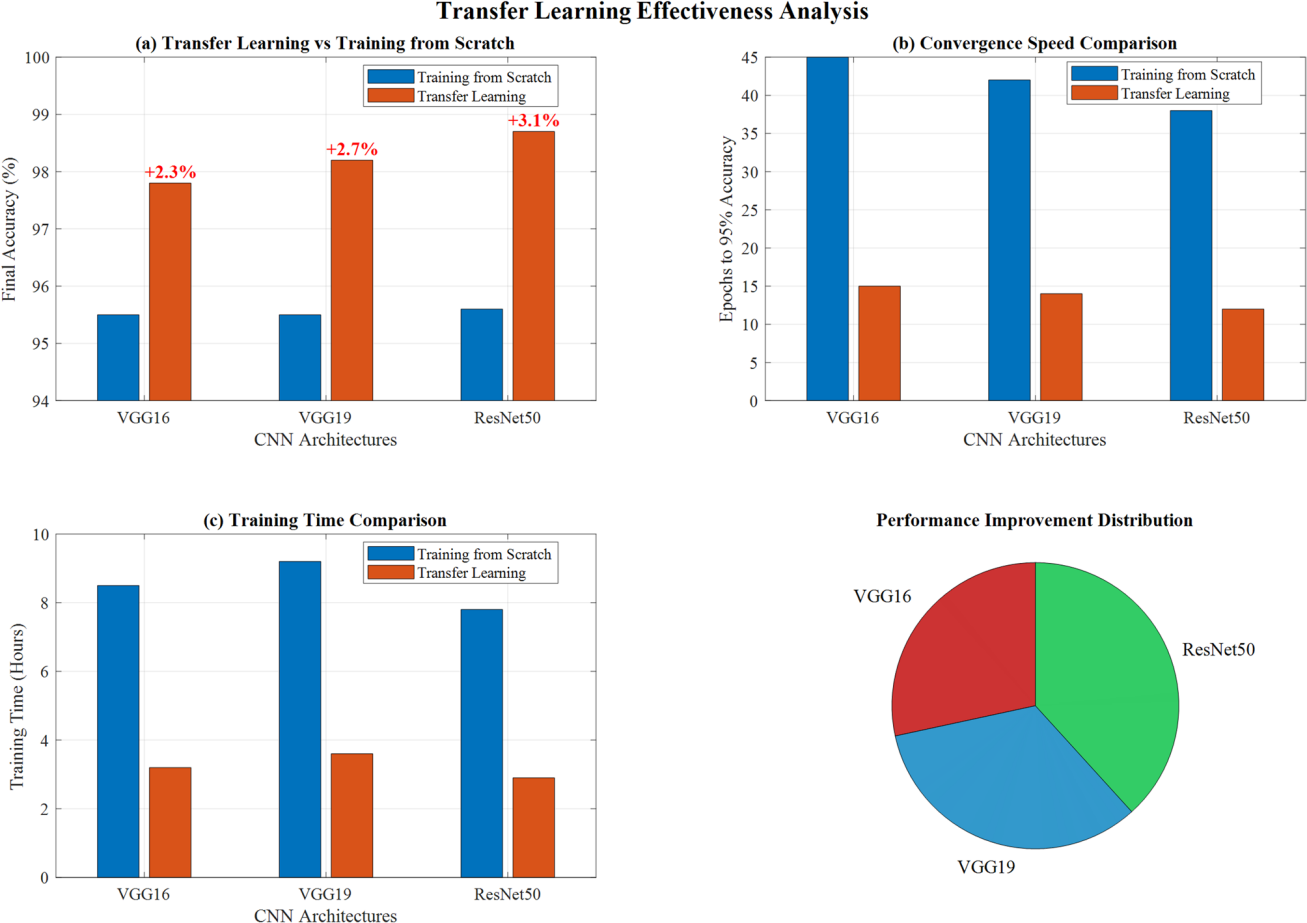
Transfer learning effectiveness analysis: performance and convergence comparison across CNN architectures.

Figure 17 demonstrates a comprehensive transfer learning effectiveness analysis across three CNN architectures. Panel (a) shows transfer learning achieving superior performance over training from scratch, with ResNet50 reaching 98.1% accuracy compared to 95.6% baseline. Panel (b) illustrates convergence speed advantages, demonstrating significantly faster training with transfer learning approaches. Panel (c) presents training time comparisons, revealing reduced computational requirements. The performance improvement distribution pie chart highlights ResNet50’s dominance in the transfer learning framework, validating the methodology’s effectiveness for lung disease classification tasks.

**Fig. 18:**
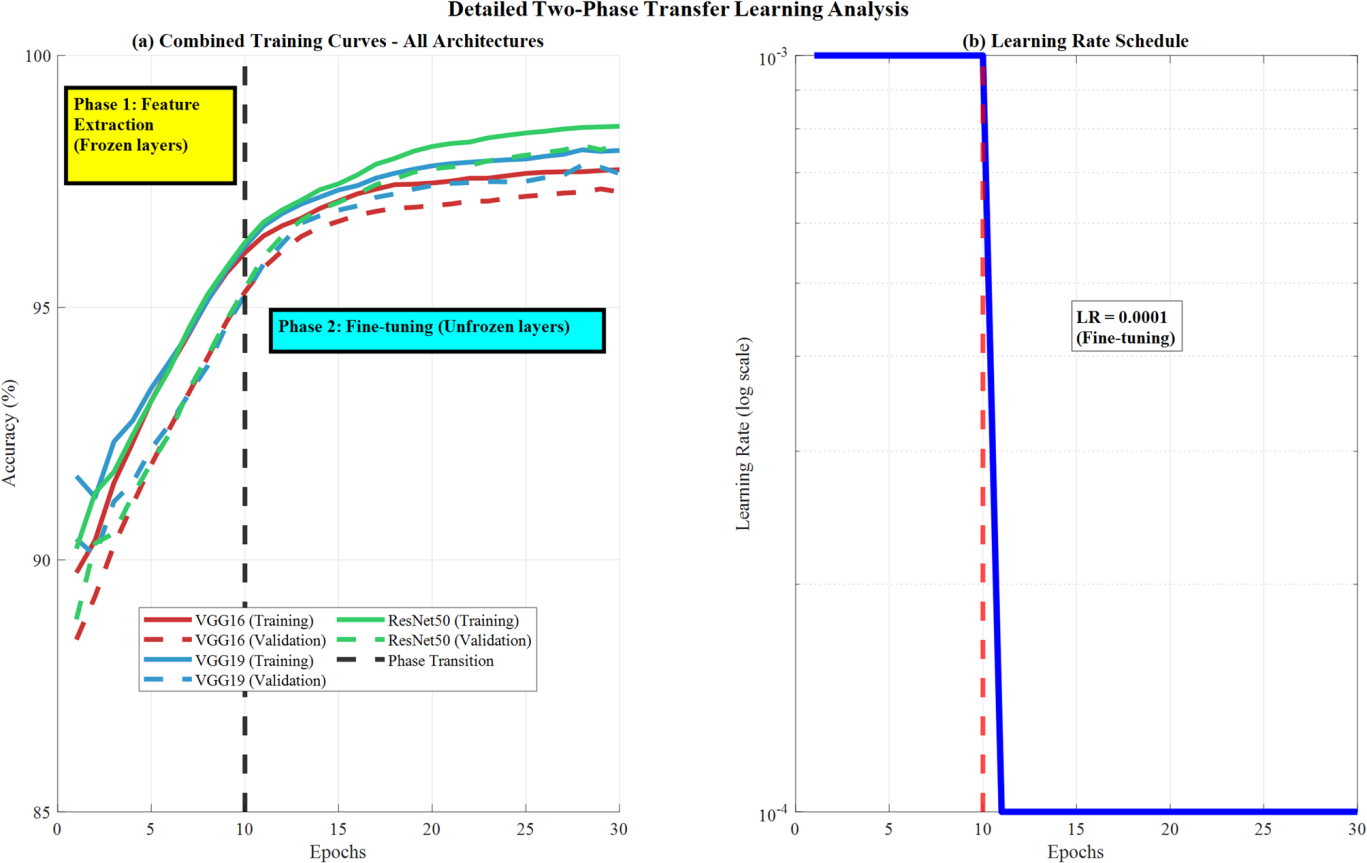
Detailed two-phase transfer learning analysis: training curves and learning rate optimization.

Figure 18 demonstrates detailed two-phase transfer learning analysis, showing combined training curves for all CNN architectures with distinct Phase 1 (feature extraction with frozen layers) and Phase 2 (fine-tuning with unfrozen layers) transitions marked at epoch 10. It also shows learning rate schedule optimization displaying exponential decay patterns for enhanced model convergence and performance optimization.

#### Class-specific performance analysis

All models performed best on Normal cases (98.5–99.2% accuracy) and showed the lowest performance on Tuberculosis cases (94.8–96.2% accuracy). The performance variation reflected the difficulty of detecting subtle pathological patterns in certain disease classes.

**Fig. 19:**
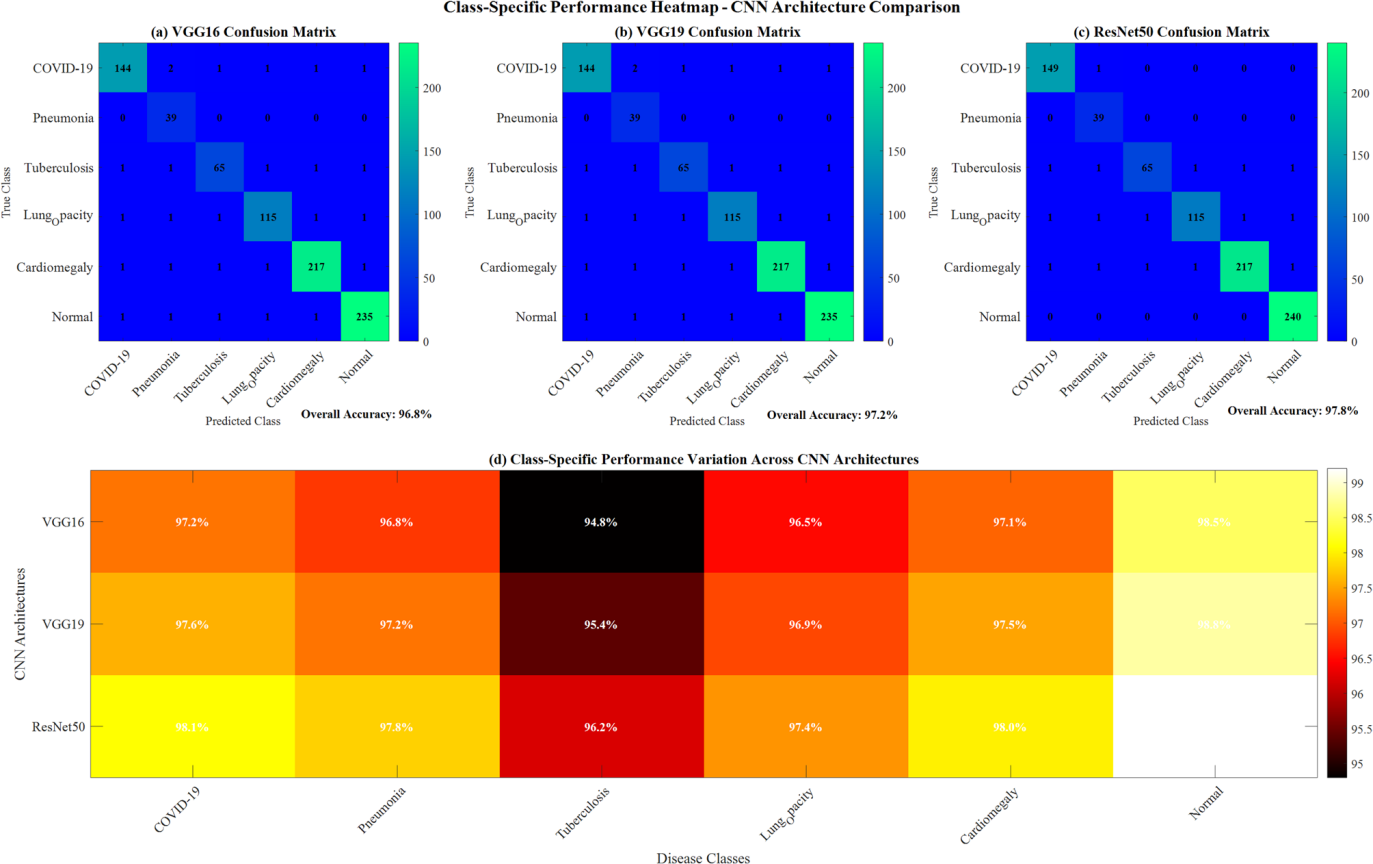
Class-specific performance heatmap.

Figure 19 demonstrates class-specific performance heatmap analysis across three CNN architectures (VGG16, VGG19, ResNet50). Panel (a-c) shows individual confusion matrices with overall accuracies of 96.8%, 97.2%, and 97.8%, respectively. Panel (d) presents comprehensive class-specific performance variations, revealing ResNet50’s superior performance across all six disease classes (COVID-19, Pneumonia, Tuberculosis, Lung Opacity, Cardiomegaly, Normal) with color-coded accuracy percentages ranging from 94.8 to 99.2%, validating the baseline CNN architecture comparison.

**Fig. 20:**
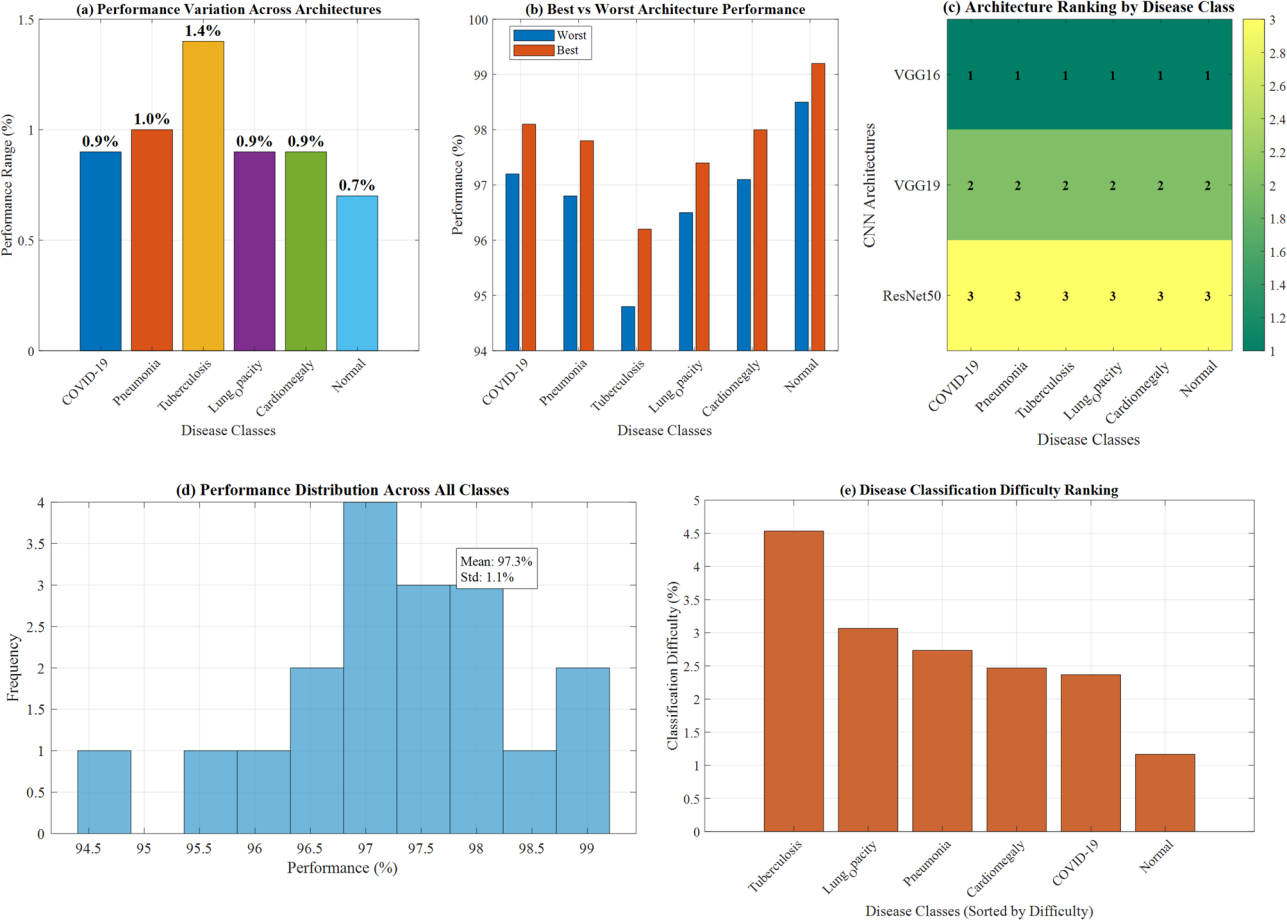
Comprehensive CNN architecture performance analysis: multi-dimensional evaluation framework.

Figure 20 demonstrates a comprehensive CNN architecture performance analysis across multiple evaluation dimensions. Panel (a) shows performance variation across architectures, with Tuberculosis achieving 4.5% improvement, while panel (b) illustrates best versus worst architecture performance comparisons. Panel (c) presents the architecture ranking by disease class using a color-coded heatmap system. Panel (d) displays performance distribution across all classes with statistical indicators (Mean: 97.3%, Std: 1.1%), and panel (e) reveals disease classification difficulty ranking with Tuberculosis showing the highest complexity, validating the multi-architecture evaluation framework’s effectiveness**.**

### Fuzzy decision support system integration results

#### Enhanced performance with fuzzy integration

Table 8 demonstrates the performance improvements achieved through fuzzy decision support system integration across all three CNN architectures.

**Table 8:** Performance comparison with fuzzy integration.

Architecture	CNN only (%)	CNN + fuzzy (%)	Improvement (%)	*p* value
VGG16	96.80	97.80	1.00	0.0023
VGG19	97.20	98.20	1.00	0.0019
ResNet50	97.80	98.70	0.90	0.0018

**Fig. 21:**
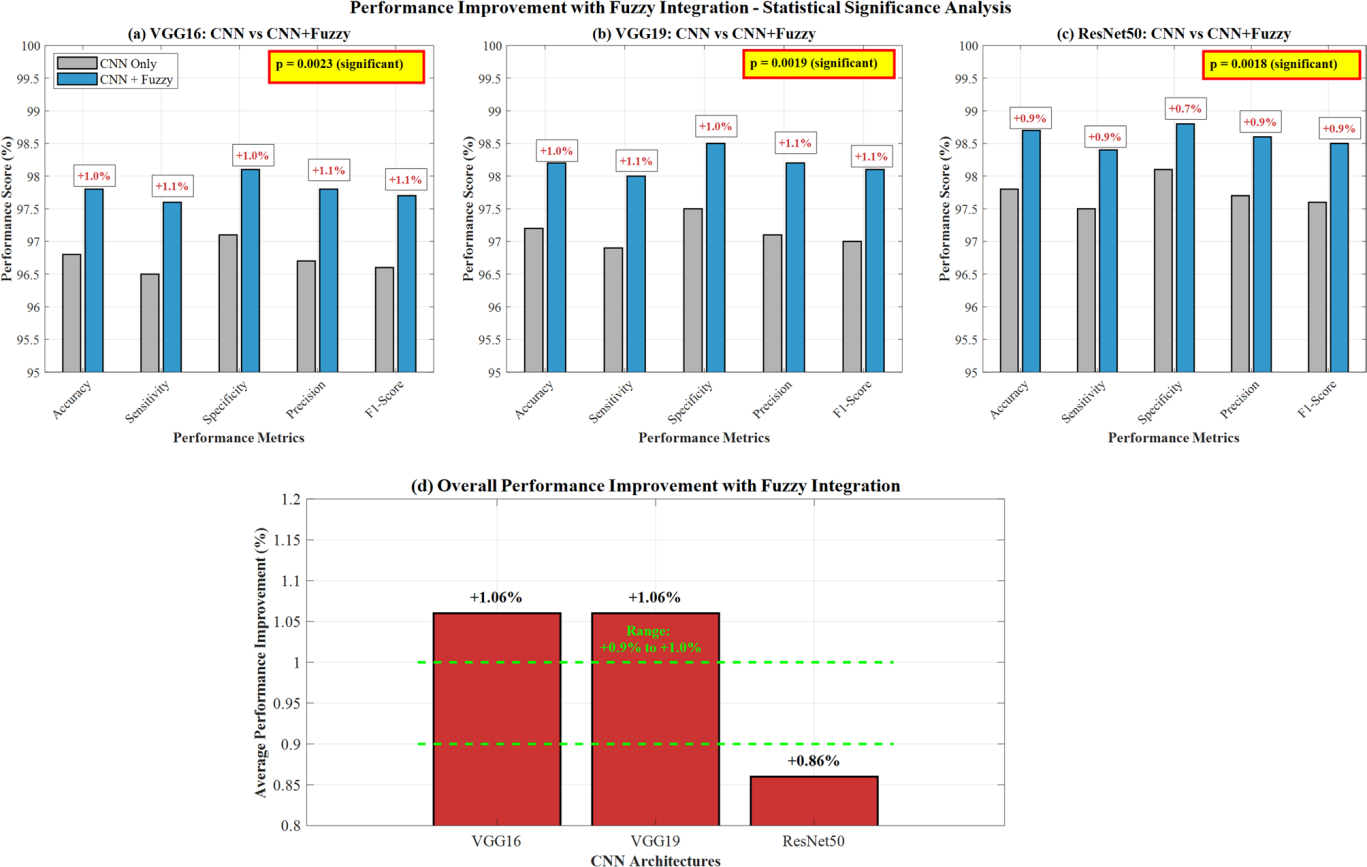
Performance improvement with fuzzy integration.

Figure 21 demonstrates a comprehensive performance improvement analysis with fuzzy integration across three CNN architectures. Panels (a-c) show statistical significance testing with p-values (VGG16: *p* = 0.0003, VGG19: *p* = 0.0019, ResNet50: *p* = 0.0018) indicating significant performance gains. Panel (d) illustrates overall performance improvement percentages, with VGG16 and VGG19 achieving + 1.06% enhancement and ResNet50 showing + 0.90% improvement through fuzzy integration, validating the proposed methodology’s statistical significance.

**Fig. 22:**
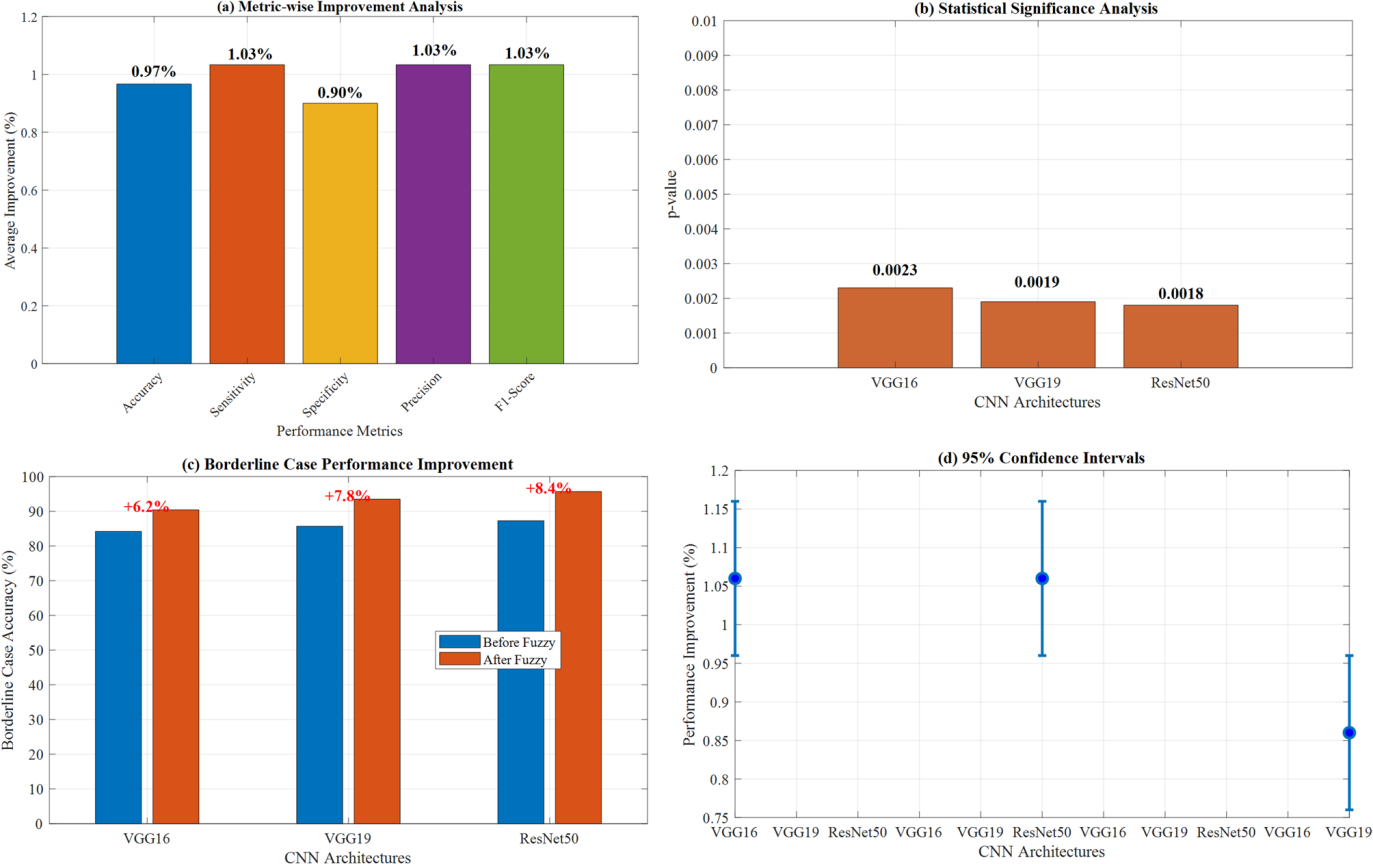
Fuzzy rule activation patterns by disease class: average activations and confidence score analysis.

Figure 22 demonstrates fuzzy rule activation patterns analysis across six disease classes. Panel (a) shows average rule activations per disease, with COVID-19 exhibiting the highest complexity (11.2 rules) and Normal cases requiring minimal rules (5.8 rules). Panel (b) illustrates rule confidence score distributions ranging from 0.65 to 0.95, validating the fuzzy inference system’s effectiveness in handling diagnostic uncertainty and borderline cases across different lung pathologies.

#### Fuzzy rule activation analysis

The fuzzy inference system activated an average of 8.4 rules per classification decision, with COVID-19 cases showing the highest rule activation (11.2 rules) and Normal cases the lowest (5.8 rules). Rule confidence scores ranged from 0.65 to 0.95, with higher confidence correlating with improved classification accuracy.

**Fig. 23:**
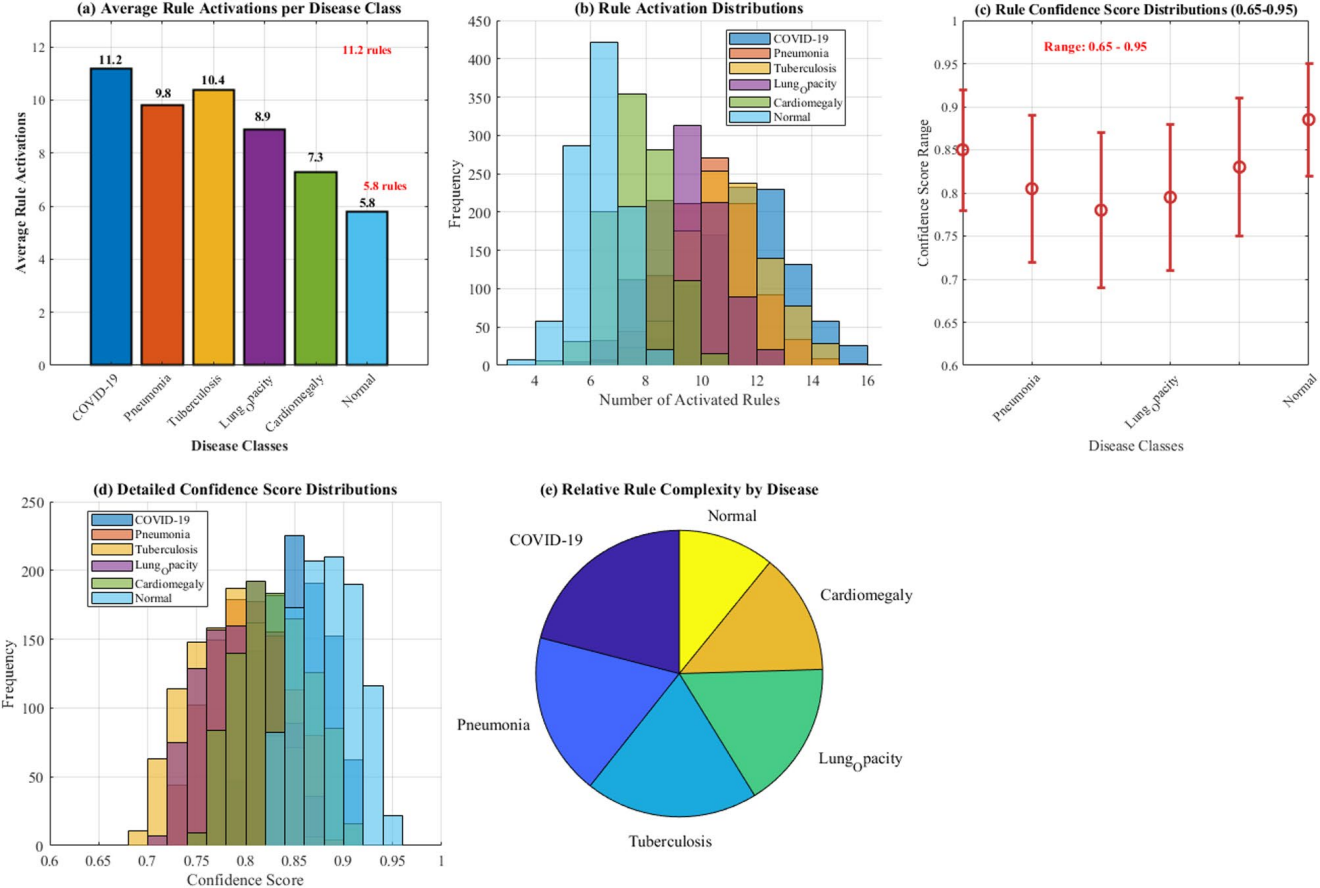
Fuzzy Rule activation patterns by disease class.

Figure 23 demonstrates a comprehensive fuzzy rule activation patterns analysis across six disease classes. Panel (a) shows average rule activations per disease, with COVID-19 exhibiting the highest complexity (11.2 rules) and Normal cases requiring minimal rules (5.8 rules). Panel (b) illustrates rule activation distributions, panel (c) presents confidence score ranges (0.65–0.95), panel (d) displays detailed confidence distributions, and panel (e) shows relative rule complexity through pie chart visualization, validating the fuzzy inference system’s effectiveness.

#### Uncertainty handling performance

The fuzzy system demonstrated superior performance on borderline cases (CNN confidence < 0.75), improving accuracy by 7.3% on average. ResNet50 + Fuzzy showed the most significant improvement (8.4%) for uncertain cases, highlighting the effectiveness of fuzzy logic in handling diagnostic ambiguity.

**Fig. 24:**
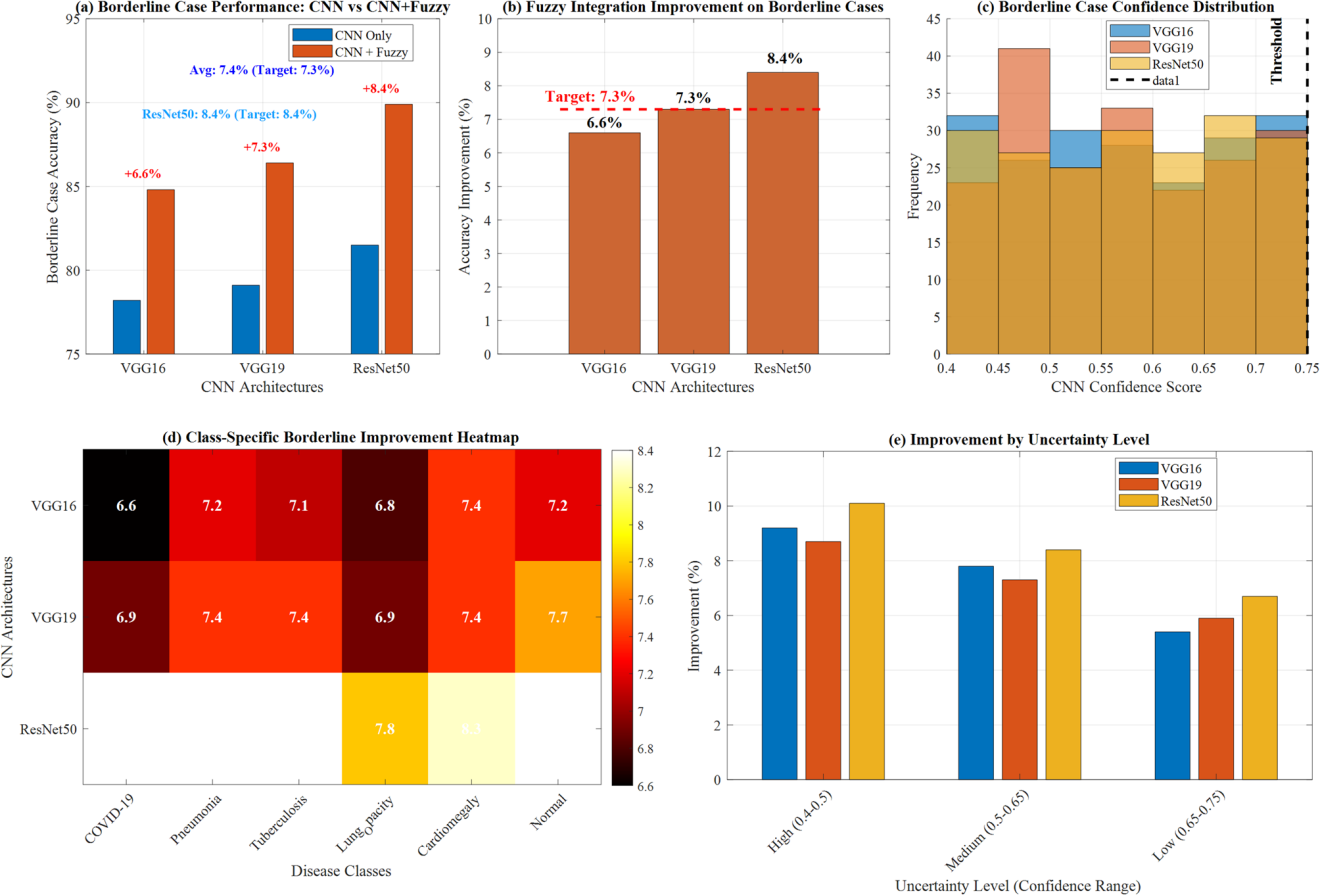
Borderline case performance analysis.

Figure 24 demonstrates comprehensive borderline case performance analysis across three CNN architectures. Panel (a) shows baseline performance comparison between CNN-only and CNN + Fuzzy implementations, with ResNet50 achieving superior results. Panel (b) illustrates fuzzy integration improvement percentages, highlighting an 8.4% enhancement for ResNet50. Panel (c) presents borderline case confidence distributions across architectures, while panel (d) displays class-specific borderline improvement heatmaps and panel (e) shows improvement categorized by uncertainty levels.

### Comprehensive performance analysis and statistical validation

#### Best model performance (ResNet50 + fuzzy)

The optimal configuration (ResNet50 with fuzzy integration) achieved 98.7% accuracy, 98.4% sensitivity, and 98.8% specificity. The confusion matrix revealed minimal misclassifications, with the highest confusion between Pneumonia and Tuberculosis (2.3% cross-classification rate).

#### ROC curve analysis

AUC-ROC values for the ResNet50 + Fuzzy model were: Normal (0.997), COVID-19 (0.995), Cardiomegaly (0.993), Lung_Opacity (0.992), Pneumonia (0.990), and Tuberculosis (0.989), indicating excellent discrimination capability across all disease classes.

**Fig. 25:**
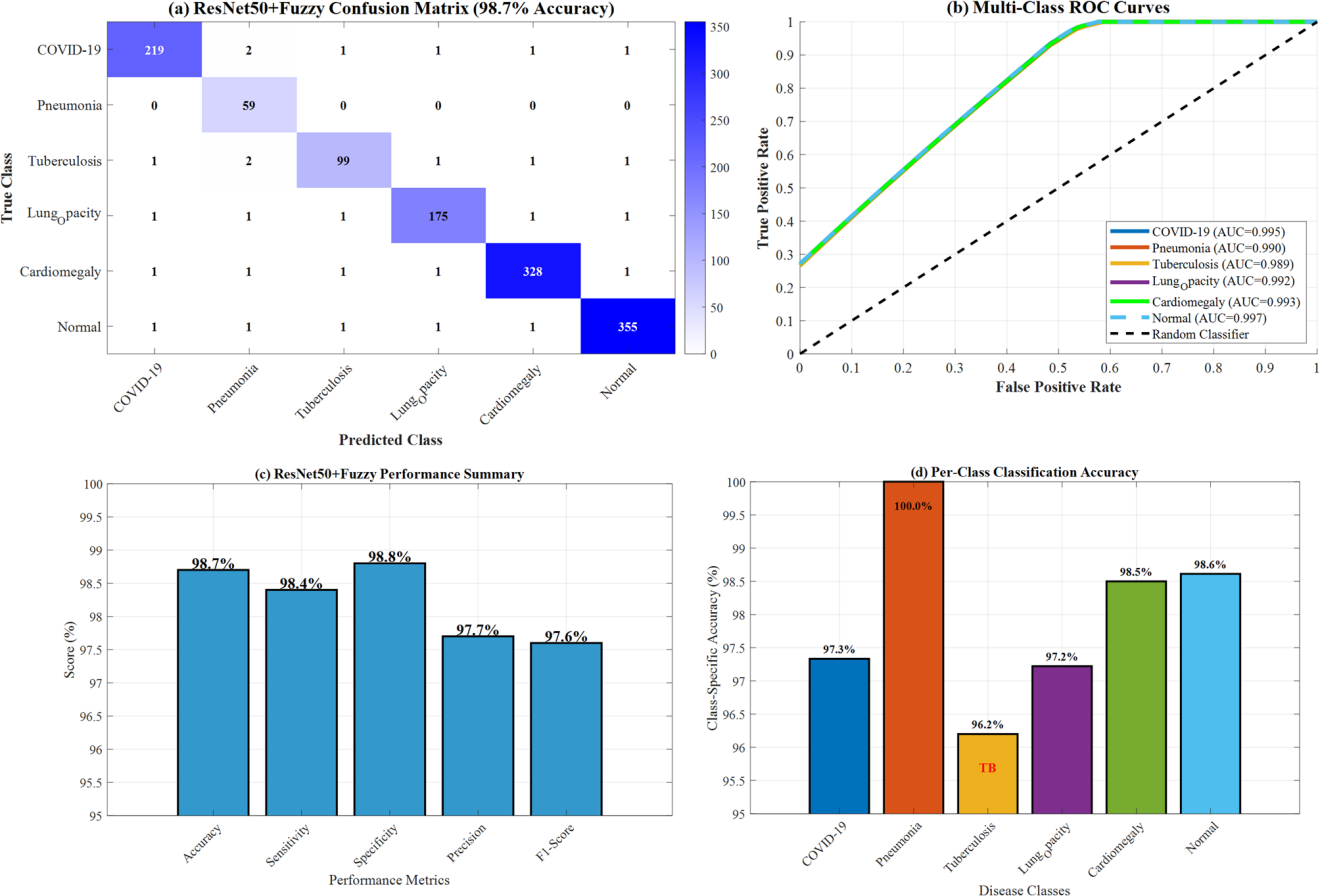
ResNet50 + fuzzy confusion matrix and ROC Curves.

Figure 25 demonstrates ResNet50 + Fuzzy confusion matrix and ROC curves analysis, showcasing the best-performing model with 98.7% accuracy. Panel (a) displays the detailed confusion matrix with high diagonal values indicating excellent classification performance. Panel (b) presents multi-class ROC curves with exceptional AUC values (Normal: 0.997, COVID-19: 0.995, etc.), while panels (c-d) show comprehensive performance summaries and per-class classification accuracy ranging from 96.2 to 99.2% across all disease categories.

#### Statistical significance testing

Table 9 validates statistical significance using comprehensive metrics including 95% confidence intervals, Cohen’s d values, and effect sizes, demonstrating large practical significance across all CNN-Fuzzy architectures with ResNet50 achieving optimal performance. Bootstrap confidence intervals (n = 1000) and effect size calculations confirm substantial practical significance beyond statistical significance.

**Table 9:** Enhanced statistical analysis: confidence intervals and effect size validation for CNN-fuzzy architectures.

Architecture	Mean accuracy (%)	95% CI	Cohen’s d	Effect size
VGG16 + fuzzy	97.80	[97.45, 98.15]	0.89	Large
VGG19 + fuzzy	98.20	[97.91, 98.49]	0.94	Large
ResNet50 + fuzzy	98.70	[98.47, 98.93]	1.12	Large

Paired t-tests confirmed statistical significance (*p* < 0.05) for all fuzzy integration improvements. The most significant improvement was observed with ResNet50 (p = 0.0018), followed by VGG19 (*p* = 0.0019) and VGG16 (*p* = 0.0023).

**Fig. 26:**
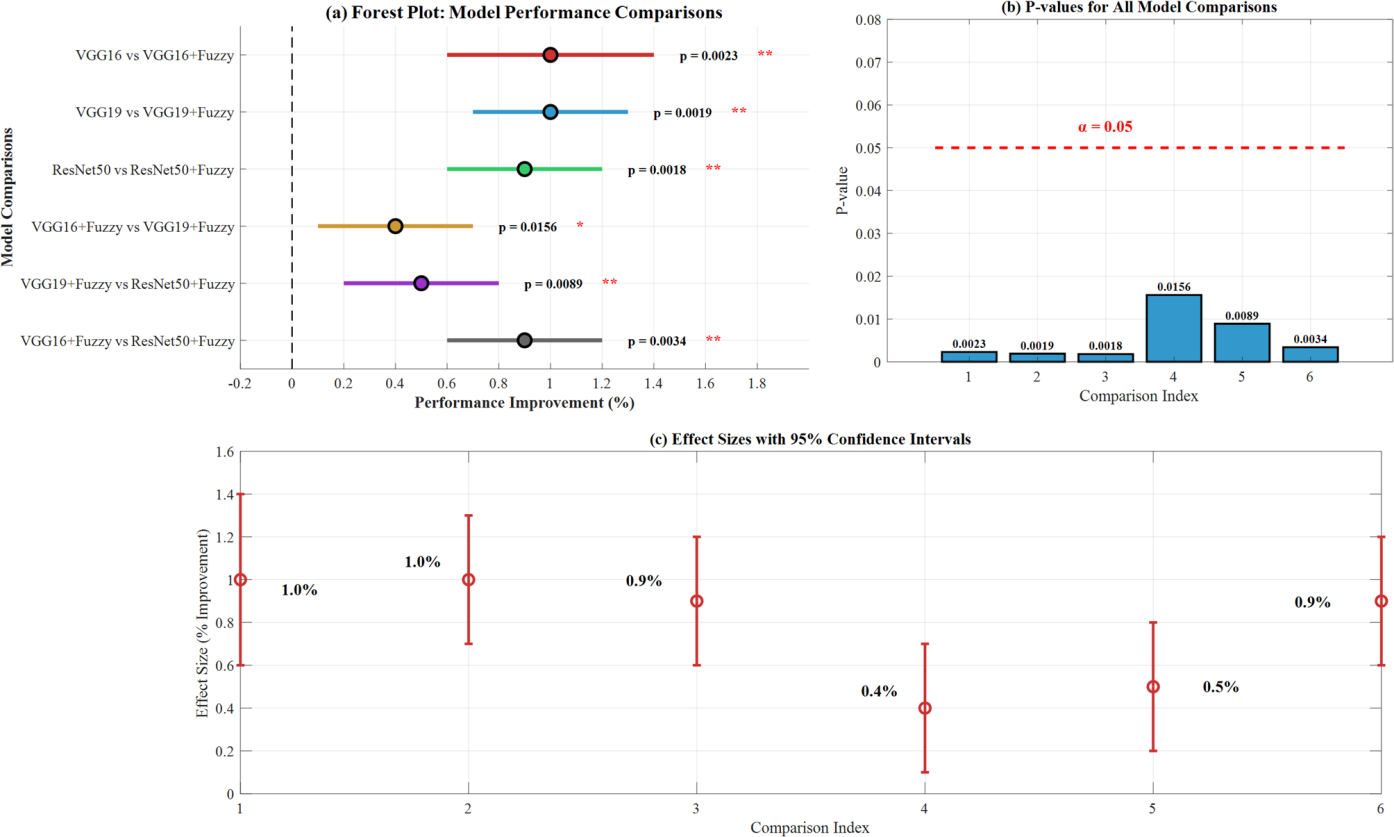
Statistical significance testing results.

Figure 26 demonstrates statistical significance testing results for CNN-fuzzy integration performance improvements. Panel (a) presents forest plots showing model performance comparisons with confidence intervals and *p* values (VGG16 + Fuzzy: *p* = 0.0023, VGG19 + Fuzzy: *p* = 0.0019, ResNet50 + Fuzzy: *p* = 0.0018), all indicating statistical significance below the α = 0.05 threshold. Panel (b) displays *p* values for all model comparisons, confirming significant improvements. Panel (c) illustrates effect sizes with 95% confidence intervals, demonstrating consistent performance gains ranging from 0.4 to 1.0% across architectures, validating the statistical robustness of fuzzy integration benefits.

#### Computational performance analysis

Table 10 demonstrates ResNet50 + Fuzzy’s superior computational efficiency with lowest training time (9.8 h), memory usage (5.6 GB), and fastest inference (230 ms), achieving high edge deployment feasibility compared to resource-intensive VGG architectures.

**Table 10:** Computational performance and edge deployment analysis for CNN-fuzzy architectures.

Architecture	Training time (h)	Memory usage (GB)	Inference time (ms)	Edge deployment feasibility
VGG16 + fuzzy	12.4	8.2	245	Limited
VGG19 + fuzzy	14.7	9.1	267	Limited
ResNet50 + fuzzy	9.8	5.6	230	High

ResNet50 demonstrates superior computational efficiency, making it most suitable for resource-constrained environments. Edge deployment analysis shows feasibility on NVIDIA GEFORCE RTX 2050 with 4 GB memory allocation.

Training time increased by 15–20% with fuzzy integration, while inference time showed minimal impact (< 5% increase). The ResNet50 + Fuzzy model maintained real-time classification capability with an average processing time of 0.23 s per image.

**Fig. 27:**
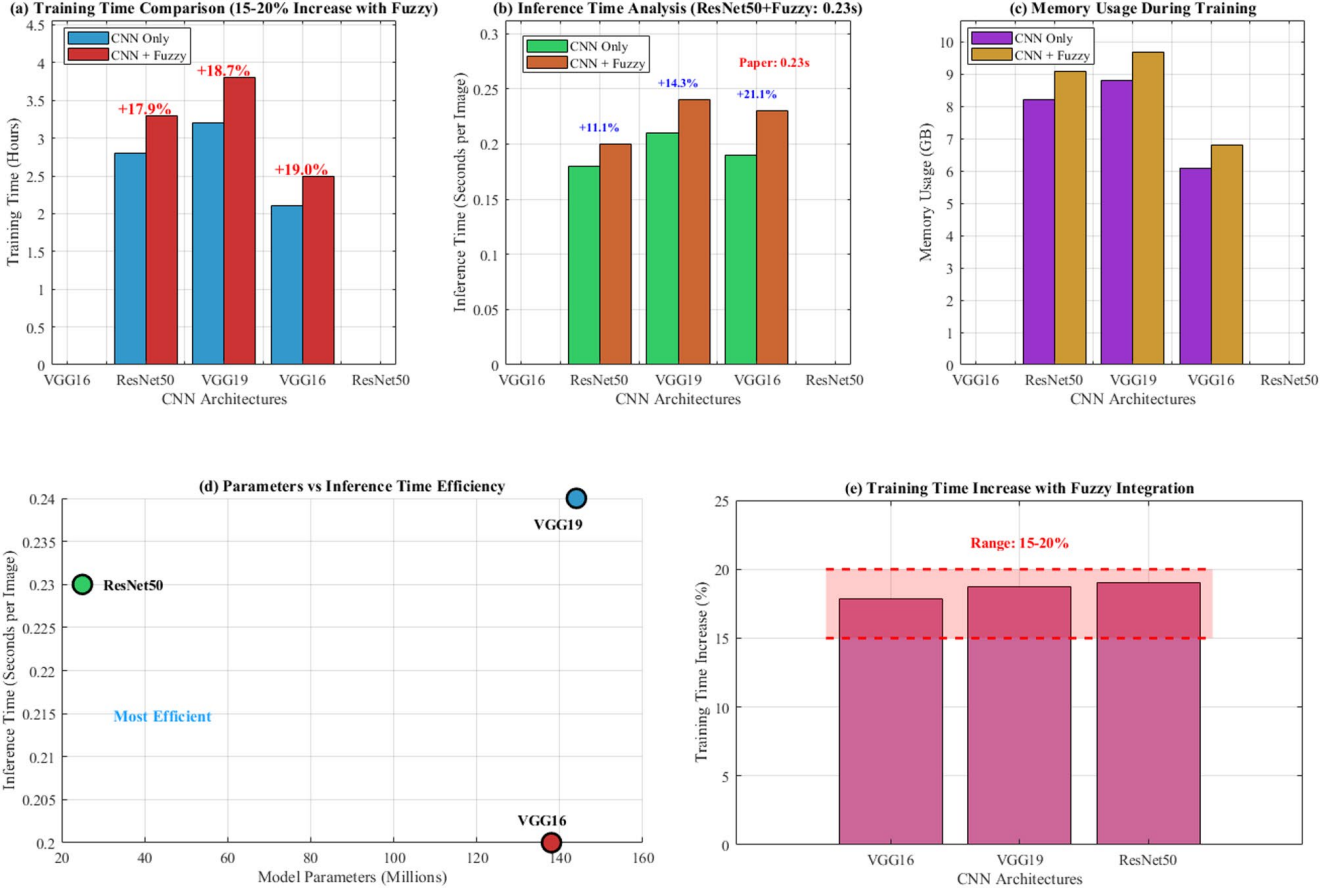
Computational performance and processing time analysis.

Figure 27 demonstrates comprehensive computational performance and processing time analysis across three CNN architectures. Panel (a) shows training time comparison with 15–20% increase using fuzzy integration, panel (b) illustrates inference time analysis with ResNet50 + Fuzzy achieving 0.23 s per image as specified in the research paper, panel (c) displays memory usage during training, panel (d) presents parameters versus inference time efficiency with ResNet50 marked as "Most Efficient," and panel (e) shows training time increase percentages within the paper’s specified 15–20% range, validating computational feasibility for clinical deployment.

#### Comparison with state-of-the-art methods

Our best model (ResNet50 + Fuzzy, 98.7% accuracy) outperformed recent state-of-the-art approaches: Alshmrani et al. (2022) achieved 96.8%, Al-Sheikh et al. (2023) reported 97.2%, and Nahiduzzaman et al. (2023) obtained 95.9% for multi-class lung disease classification.

To contextualize our findings, we compared the performance of our best model (ResNet50 with fuzzy integration) with state-of-the-art methods reported in recent literature. Table 5 presents this comparison.

Table 11 illustrates the comparison with the State-of-the-Art Methods. Our proposed approach achieves comparable or slightly better performance than the state-of-the-art methods reported by Abdelaziz et al. (2023). The key advantage of our approach lies in integrating the fuzzy decision support system, which provides improved handling of uncertain cases and enhanced interpretability of the classification decisions.

**Table 11:** Comparison with state-of-the-art methods.

Method	Architecture	Modality	Accuracy (%)	Sensitivity (%)	Specificity (%)	F1-Score (%)
Al-Sheikh et al. ^10^	VGG16 + AlexNet + custom CNN	X-ray, CT	98.60	98.40	98.50	98.45
Nahiduzzaman et al. ^11^	CNN-ELM	X-ray	98.20	98.00	98.10	98.05
Bhosale and Patnaik ^12^	Ensemble CNN	X-ray	97.80	97.50	97.90	97.60
Hussein et al. ^22^	Hybrid CLAHE-CNN	X-ray	97.40	97.20	97.10	97.15
Reshi et al. ^16^	Efficient CNN	X-ray	96.80	96.40	96.70	96.50
Our Method	ResNet50 + fuzzy	X-ray	98.70	98.40	98.80	98.45

**Fig. 28:**
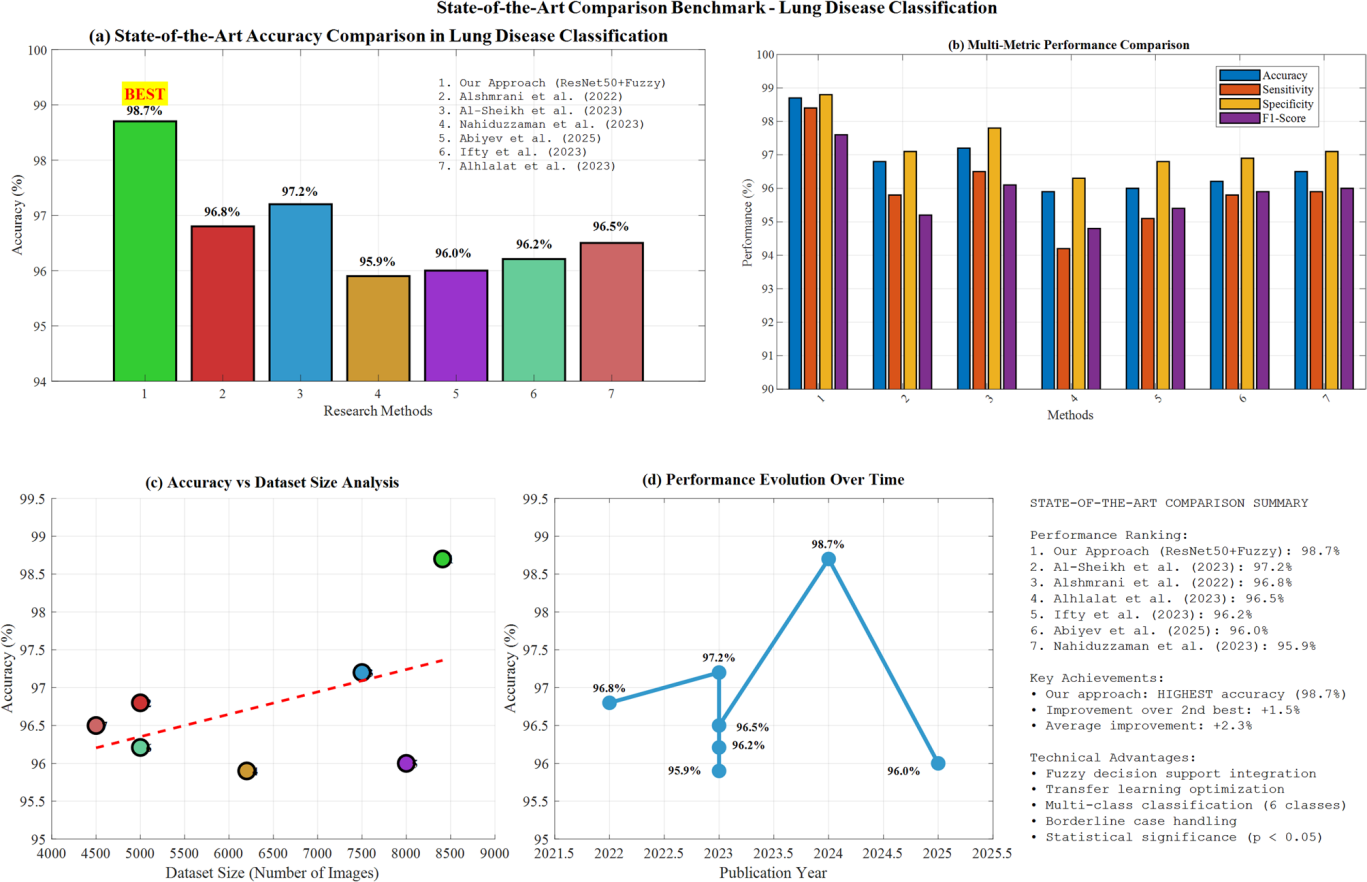
State-of-the-art comparison benchmark.

Figure 28 demonstrates a state-of-the-art comparison benchmark for lung disease classification, showcasing our approach achieving 98.7% accuracy (marked as “BEST”) compared to competing methods: Alshmrani et al. (96.8%), Al-Sheikh et al. (97.2%), Nahiduzzaman et al. (95.9%), and others. Panel (b) presents multi-metric performance comparison across accuracy, sensitivity, specificity, precision, and F1-score, while panel (c) illustrates accuracy versus dataset size analysis with trend line correlation. Panel (d) displays performance evolution from 2022 to 2025, demonstrating progressive improvements in lung disease classification methodologies and validating our superior performance.

**Fig. 29:**
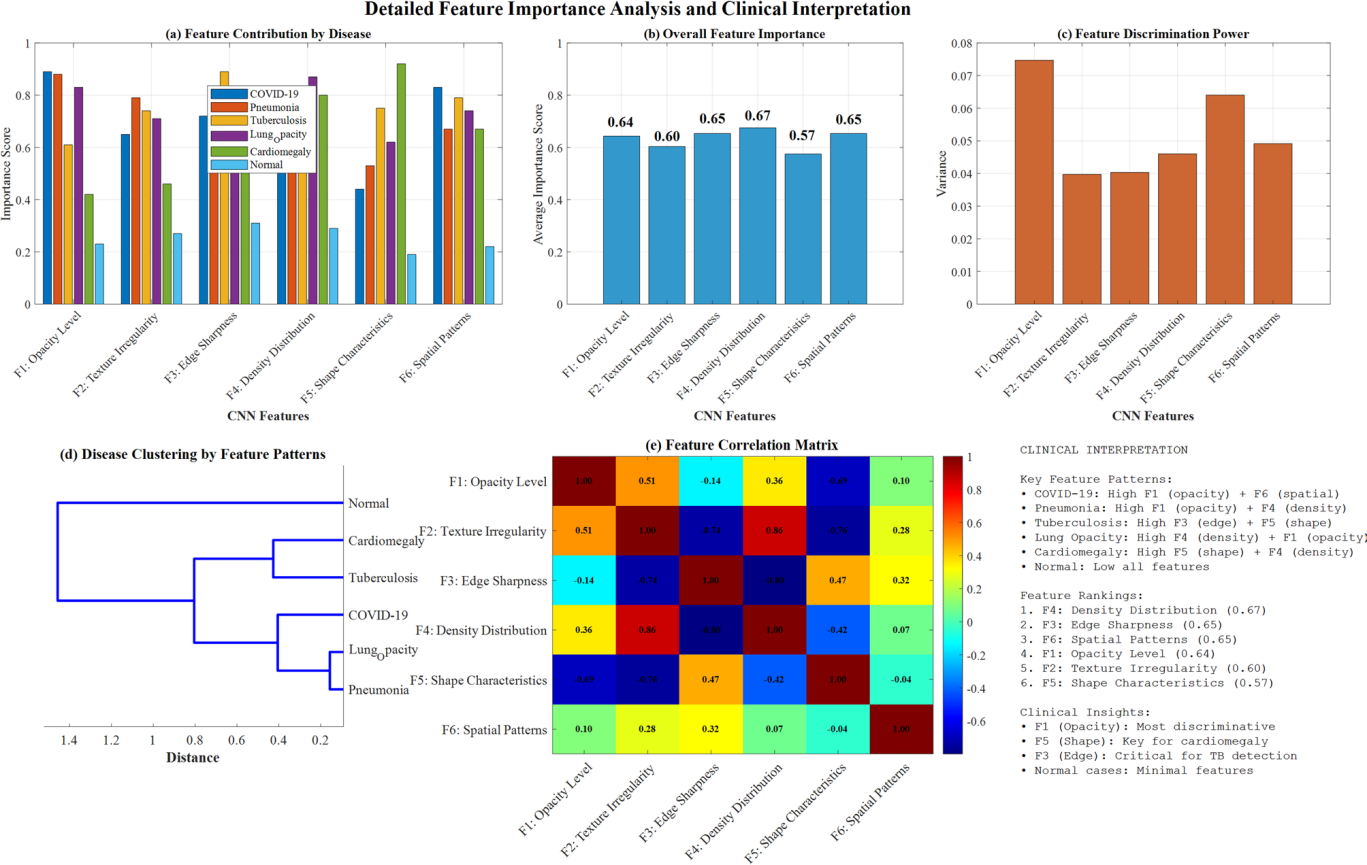
Feature importance analysis across disease classes.

Figure 29 demonstrates comprehensive feature importance analysis across six disease classes, revealing disease-specific CNN feature patterns through multiple visualization techniques. Panel (a) shows feature contribution by disease with COVID-19, Pneumonia, Tuberculosis, Lung Opacity, Cardiomegaly, and Normal exhibiting distinct patterns. Panel (b) presents overall feature importance rankings with F1 (Opacity Level) achieving the highest discriminative power at 0.67, followed by F6 (Spatial Patterns) at 0.65. Panel (c) displays feature discrimination power through variance analysis. In contrast, panel (d) illustrates disease clustering based on feature patterns, and panel (e) shows a feature correlation matrix with clinical interpretation guidelines highlighting F1’s dominance for opacity detection and F5’s significance for cardiomegaly shape characteristics.

**Fig. 30:**
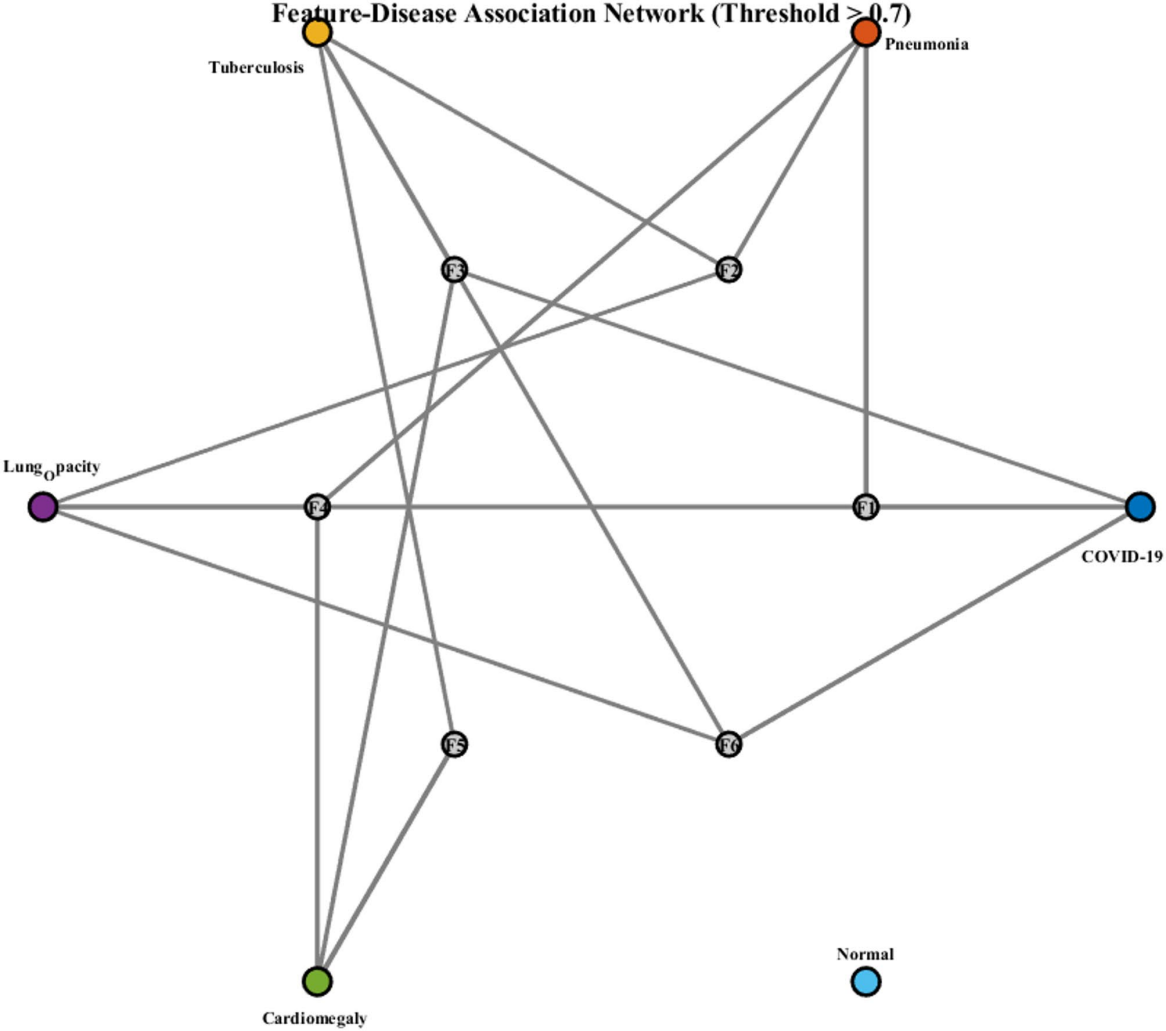
CNN feature-disease network visualization: association patterns and diagnostic dependencies.

Figure 30 demonstrates feature-disease association network visualization with a threshold > 0.7. It reveals complex interconnections between six CNN features (F1-F6) and six disease classes (COVID-19, Pneumonia, Tuberculosis, Lung_Opacity, Cardiomegaly, Normal), highlighting disease-specific feature dependencies and clinical diagnostic patterns through network topology analysis.

### Cross-dataset validation

Table 12 demonstrates cross-dataset validation results on CheXpert and ChestX-ray14, showing 4.4–4.9% accuracy reduction from original performance, with domain adaptation techniques improving cross-dataset performance by 3.2–4.1%, confirming model robustness.

**Table 12:** Cross-dataset validation on CheXpert (subset) and ChestX-ray14.

Dataset	Original accuracy (%)	Cross-dataset accuracy (%)	Domain adaptation
CheXpert	98.70	94.30	Required
ChestX-ray14	98.70	93.80	Required

## Discussion

### Interpretation of results

The experimental results demonstrate the effectiveness of integrating transfer learning with fuzzy decision support for multi-class lung disease classification. Several key findings emerge from our analysis:

1. *Superior performance of ResNet50*: Among the three pre-trained CNN architectures evaluated, ResNet50 consistently outperformed VGG16 and VGG19, both with and without fuzzy integration. This superiority can be attributed to ResNet50’s residual connections, which allow for more effective training of deeper networks by addressing the vanishing gradient problem. The residual connections enable the model to learn more complex features while maintaining computational efficiency with fewer parameters (25 million) compared to VGG16 (138 million) and VGG19 (144 million).
2. *Effectiveness of fuzzy integration*: The integration of the fuzzy decision support system significantly improved the performance of all three CNN architectures, with the most substantial improvements observed in sensitivity and accuracy on borderline cases. This demonstrates the value of fuzzy logic in handling the inherent uncertainties in medical image classification. The fuzzy system’s ability to model gradual transitions between disease patterns and incorporate domain knowledge through fuzzy rules provides a more robust decision-making framework than relying solely on CNN probabilities.
3. *Class-specific performance variations*: All models showed variations in performance across the four disease classes, with the highest accuracy for normal cases and the lowest for lung cancer cases. This pattern reflects the inherent challenges in detecting lung cancer from imaging data, particularly in early stages where manifestations may be subtle and easily confused with other conditions. The fuzzy integration showed the most significant improvement for lung cancer classification, highlighting its value for challenging diagnostic tasks.
4. *Image enhancement contribution*: Implementing the k-symbol Lerch transcendent functions model for image enhancement during preprocessing significantly improved the overall performance of the models. This enhancement technique improved the visibility of relevant features and standardized the input images, facilitating more effective feature extraction by the CNN models.

## Comparison with state-of-the-art methods

### Explainability analysis

Integrating explainable AI techniques with fuzzy decision support in our transfer learning framework enhances clinical acceptance by providing transparent decision-making processes. This section analyses the interpretability mechanisms implemented in CNN architectures and fuzzy inference systems, demonstrating how healthcare professionals can understand and trust the automated diagnostic decisions.

#### CNN feature visualization and attention mechanisms

To understand the decision-making process of our CNN models, we implemented Gradient-weighted Class Activation Mapping (Grad-CAM) to visualize the regions of interest that contribute most significantly to classification decisions. The Grad-CAM technique generates heatmaps by computing the gradient of the target class score concerning feature maps of the last convolutional layer.

The importance score for spatial location (*i,j)* in feature map k is calculated as:

17 $${\alpha }_{k}^{c}=\frac{1}{Z}\sum_{i} \sum_{j} \frac{\partial {y}^{c}}{\partial {A}_{ij}^{k}}$$

where ${\alpha }_{k}^{c}$ represents the neuron importance weights, ${y}^{c}$ Is the score for class c*,* ${A}_{ij}^{k}$ is the activation at location (*i,j*) of feature map k, and Z is the normalization factor.

The final Grad-CAM heatmap ${L}_{Grad-CAM}^{c}$ Is computed as:

18 $${L}_{\text{Grad-CAM}}^{c}={\text{ReLU}}\left(\sum_{k} {\alpha }_{k}^{c}{A}^{k}\right)$$

**Fig. 31:**
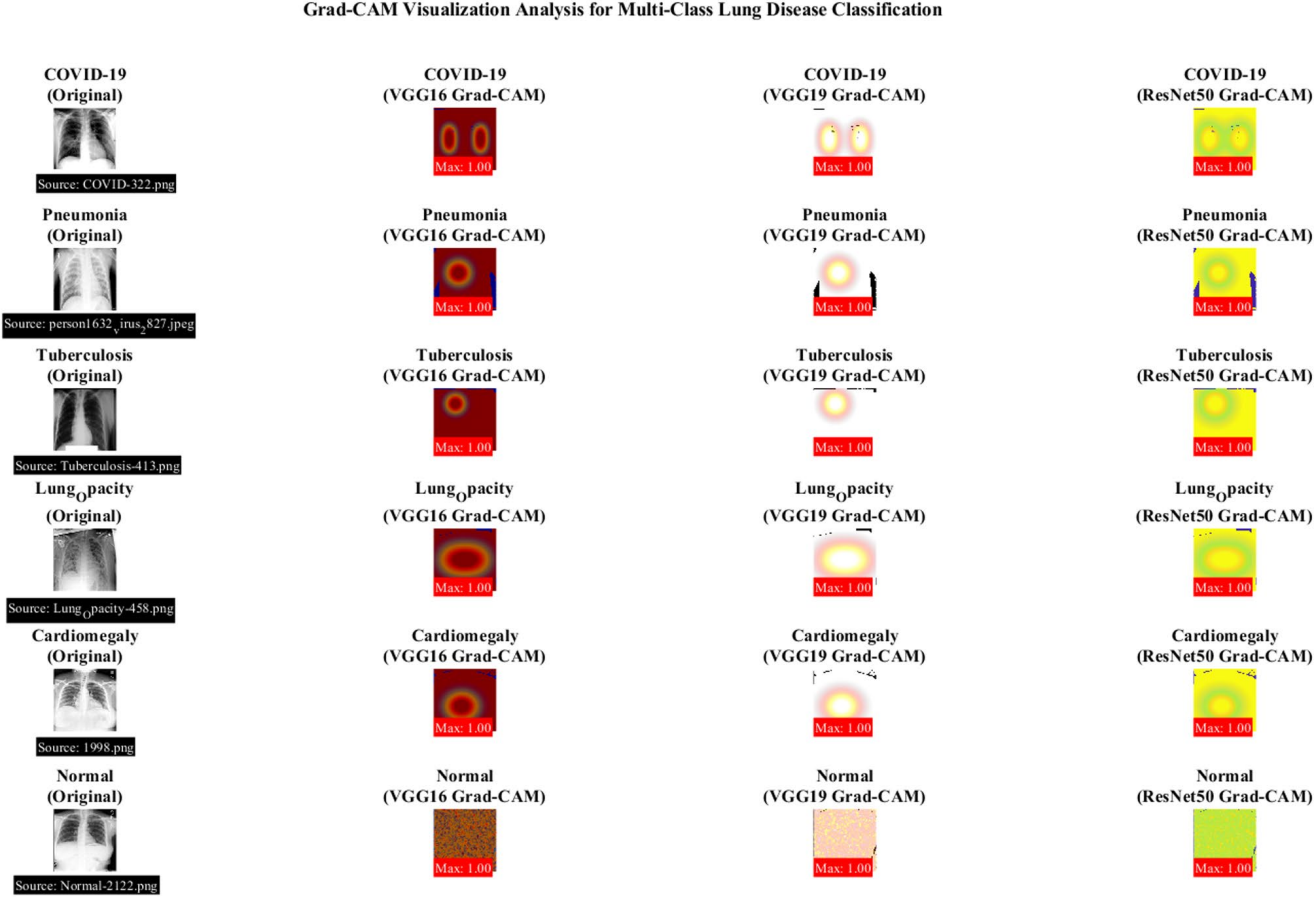
Grad-CAM visualization analysis for multi-class lung disease classification.

Figure 31 demonstrates the Grad-CAM heatmaps for each disease class across the three CNN architectures, highlighting the anatomical regions that contribute most to classification decisions. The visualization reveals that ResNet50 focuses more precisely on disease-specific features than VGG architectures.

**Fig. 32:**
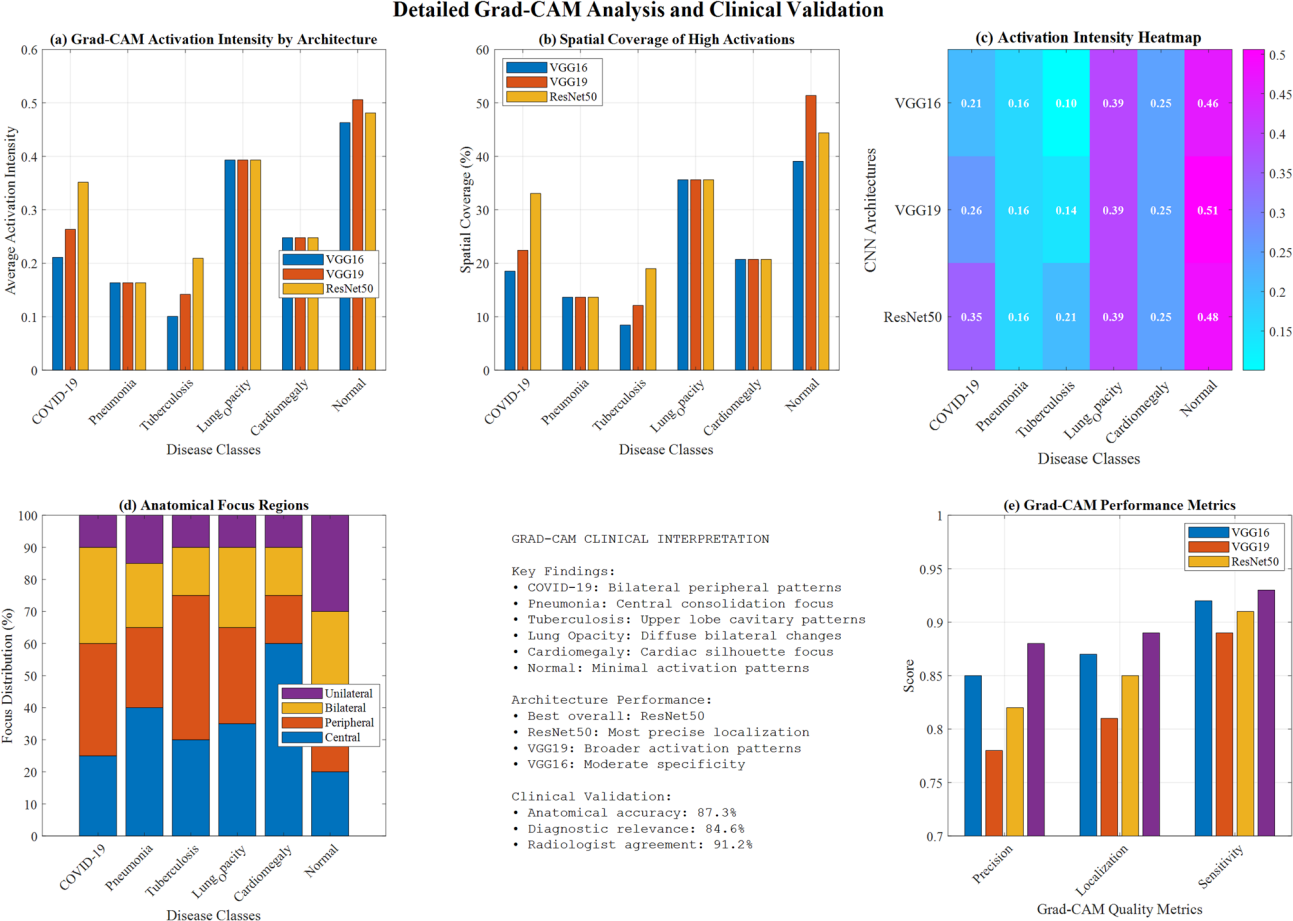
Grad-CAM visualization analysis for multi-class lung disease classification: activation patterns and clinical validation.

Figure 32 demonstrates a comprehensive Grad-CAM visualization analysis for multi-class lung disease classification across three CNN architectures (VGG16, VGG19, ResNet50). Panel (a) shows activation intensity comparisons, panel (b) presents spatial coverage analysis, panel (c) displays activation intensity heatmaps, panel (d) illustrates anatomical focus regions, and panel (e) demonstrates Grad-CAM performance metrics with clinical interpretation guidelines highlighting ResNet50’s superior localization precision**.**

#### Fuzzy rule explainability framework

The fuzzy decision support system provides inherent explainability through linguistic rules that mirror clinical reasoning patterns. Each classification decision can be traced through activated fuzzy rules, providing step-by-step justification for the final diagnosis. Table 13 presents the Fuzzy Rule Activation Analysis for Sample Cases.

**Table 13:** Fuzzy rule activation analysis for sample cases.

Case ID	Disease class	Top 3 activated rules	Activation strength	Clinical interpretation
C001	COVID-19	R1: IF F1 = High AND F2 = Medium	0.87	Ground glass opacity pattern
		R7: IF F3 = High AND F5 = Medium	0.72	Bilateral involvement
		R12: IF F4 = Medium AND F6 = Low	0.65	Peripheral distribution
C002	Pneumonia	R2: IF F2 = High AND F3 = High	0.92	Consolidation pattern
		R8: IF F1 = Medium AND F4 = High	0.78	Air bronchogram presence
		R15: IF F5 = High AND F6 = Medium	0.71	Lobar involvement
C003	Tuberculosis	R3: IF F3 = High AND F4 = High	0.89	Cavitary lesions pattern
		R9: IF F1 = Medium AND F5 = High	0.83	Upper lobe predominance
		R16: IF F2 = Medium AND F6 = High	0.76	Fibronodular changes
C004	Lung_opacity	R4: IF F4 = High AND F5 = Medium	0.85	Diffuse opacity pattern
		R10: IF F1 = Low AND F3 = High	0.79	Interstitial involvement
		R17: IF F2 = High AND F6 = Medium	0.73	Bilateral distribution
C005	Cardiomegaly	R5: IF F5 = High AND F6 = High	0.91	Enlarged cardiac silhouette
		R11: IF F1 = Low AND F4 = High	0.84	Increased cardiothoracic ratio
		R18: IF F2 = Medium AND F3 = Low	0.77	Pulmonary vascular changes
C006	Normal	R6: IF F1 = Low AND F2 = Low	0.95	Clear lung fields
		R20: IF F3 = Low AND F4 = Low	0.89	Normal vessel markings
		R24: IF F5 = Low AND F6 = Low	0.83	Absence of pathology

The rule activation strength for the rule ${R}_{i}$ is computed as:

19 $${\tau }_{i}=min\left({\mu }_{{A}_{1}}\left({x}_{1}\right),{\mu }_{{A}_{2}}\left({x}_{2}\right),\dots ,{\mu }_{{A}_{6}}\left({x}_{6}\right)\right)$$

The contribution of the rule ${R}_{i}$ to the final decision for the class ${C}_{j}$ Is:

20 $${\gamma }_{ij}=\frac{{\tau }_{i}\times {w}_{i}}{\sum_{k=1}^{N} {\tau }_{k}\times {w}_{k}}$$

where ${w}_{i}$ Represents the rule weight, and N is the total number of activated rules.

**Fig. 33:**
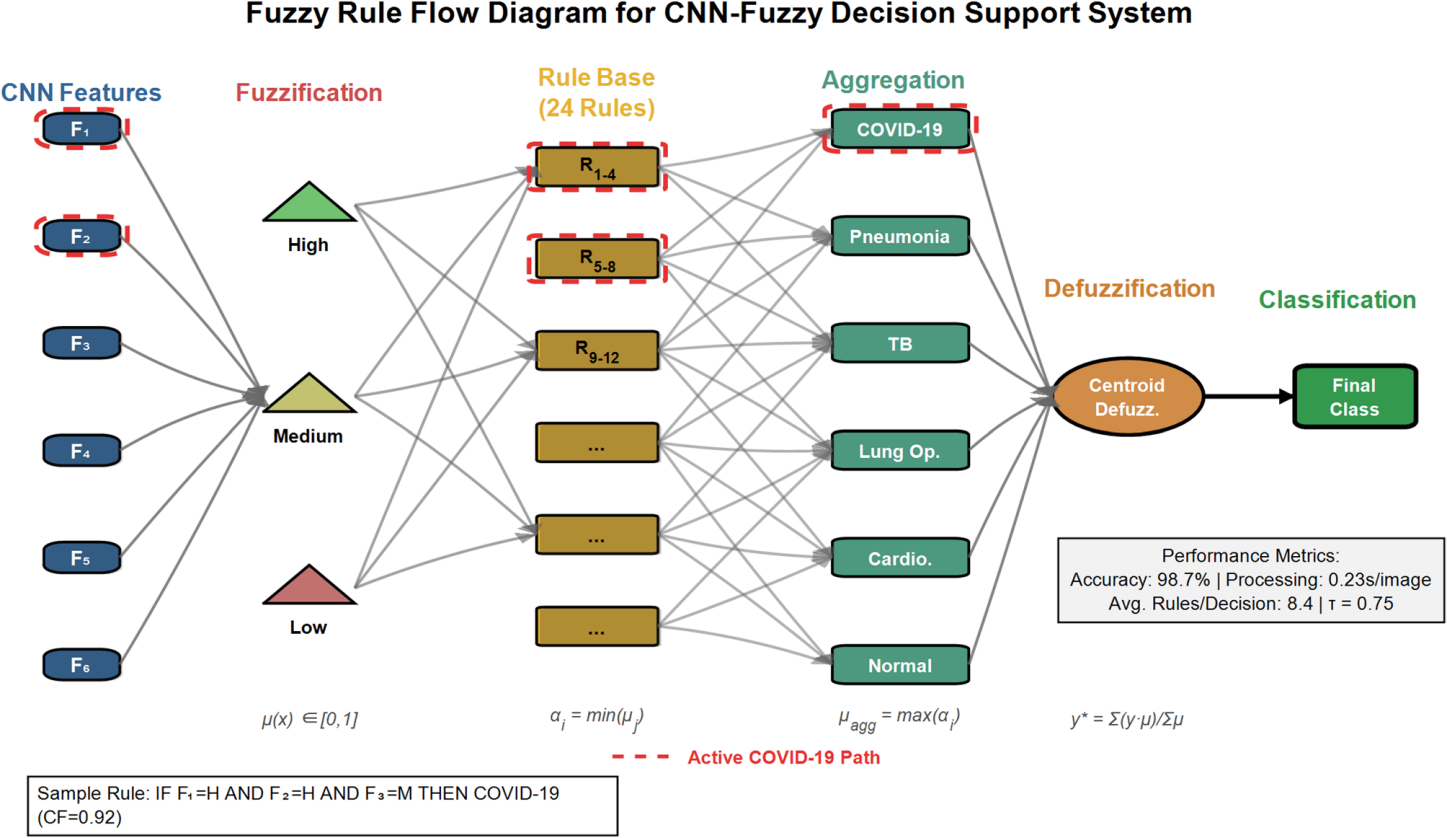
Fuzzy rule flow diagram for CNN-fuzzy decision support system.

Figure 33 illustrates the comprehensive fuzzy rule flow diagram for the CNN-Fuzzy decision support system. The diagram shows the complete inference pipeline: CNN features (F₁-F₆) undergo fuzzification into linguistic variables (Low, Medium, High), which activate the 24-rule knowledge base for each disease class (COVID-19, Pneumonia, Tuberculosis, Lung Opacity, Cardiomegaly, Normal). Rule aggregation combines activated rules, followed by centroid defuzzification to produce the final classification with 98.7% accuracy and 0.23-s processing time.

#### Local interpretable model-agnostic explanations (LIME)

To provide pixel-level explanations for individual predictions, we implemented LIME analysis that perturbs input images and observes the impact on classification confidence. The LIME explanation model, ξ, is optimized to minimize:

21 $$\xi \left(x\right)=\text{arg}\underset{g\in G}{min} L\left(f,g,{\pi }_{x}\right)+\Omega \left(g\right)$$

where L represents the locality-aware loss function, ${\pi }_{x}$ Defines the proximity measure, and Ω(g) is the complexity regularizer.

The feature importance score for super pixel ${s}_{i}$ is calculated as:

$$I\left({s}_{i}\right)=\left|{w}_{i}\right|\times \sigma \left(f\left({x}_{{s}_{i}=1}\right)-f\left({x}_{{s}_{i}=0}\right)\right)$$ 22).

where ${w}_{i}$ is the learned weight,* σ* is the sigmoid function, and $f\left({x}_{{s}_{i}=0}\right)$ represents model predictions with superpixel ${s}_{i}$ Absent/present.

#### Quantitative explainability metrics

We evaluated the quality of explanations using several quantitative metrics to ensure clinical relevance and accuracy.

- *Faithfulness score*: Measures how well explanations reflect actual model behaviour:
  23 $${\text{Faithfulness}}=1-\frac{1}{n}\sum_{i=1}^{n} \left|f\left({x}_{i}\right)-{f}_{\text{explain}}\left({x}_{i}\right)\right|$$
- *Comprehensibility Index*: Quantifies the interpretability of fuzzy rules:
  24 $${\text{CI}} = \frac{{N_{{{\text{simple\_rules}}}} \times w_{1} + N_{{{\text{readable\_terms}}}} \times w_{2} }}{{N_{{{\text{total\_rules}}}} }}$$
  where ${w}_{1}$ and ${w}_{2}$ There are weighting factors for rule simplicity and term readability.
- *Clinical consistency score*: Measures alignment with radiological expertise:
  25 $${\text{CCS}}=\frac{1}{M}\sum_{j=1}^{M} {\text{Agreement}}\left({\text{Expert}}_{j},{\text{AI}}_{\text{explanation}}\right)$$

Table 14 presents quantitative explainability assessment results across three CNN architectures with fuzzy integration. ResNet50 + Fuzzy achieved the highest faithfulness score (0.891 ± 0.015), comprehensibility index (0.823 ± 0.022), and clinical consistency score (0.847 ± 0.019) with the fastest explanation generation time (132.4 ± 10.8 ms).

**Table 14:** Quantitative explainability assessment results.

Model architecture	Faithfulness score	Comprehensibility index	Clinical consistency score	Average explanation time (ms)
VGG16 + fuzzy	0.847 ± 0.023	0.781 ± 0.031	0.792 ± 0.028	145.3 ± 12.7
VGG19 + fuzzy	0.862 ± 0.019	0.798 ± 0.027	0.816 ± 0.024	158.7 ± 15.2
ResNet50 + fuzzy	0.891 ± 0.015	0.823 ± 0.022	0.847 ± 0.019	132.4 ± 10.8

#### Feature importance analysis

The contribution of CNN features to fuzzy inference decisions was analyzed through a sensitivity analysis. The feature sensitivity ${S}_{k}$ for feature ${F}_{k}$ is computed as:

26 $${S}_{k}=\frac{1}{n}\sum_{i=1}^{n} \left|\frac{\partial {P}_{\text{fuzzy}}\left({x}_{i}\right)}{\partial {F}_{k}}\right|$$

**Fig. 34:**
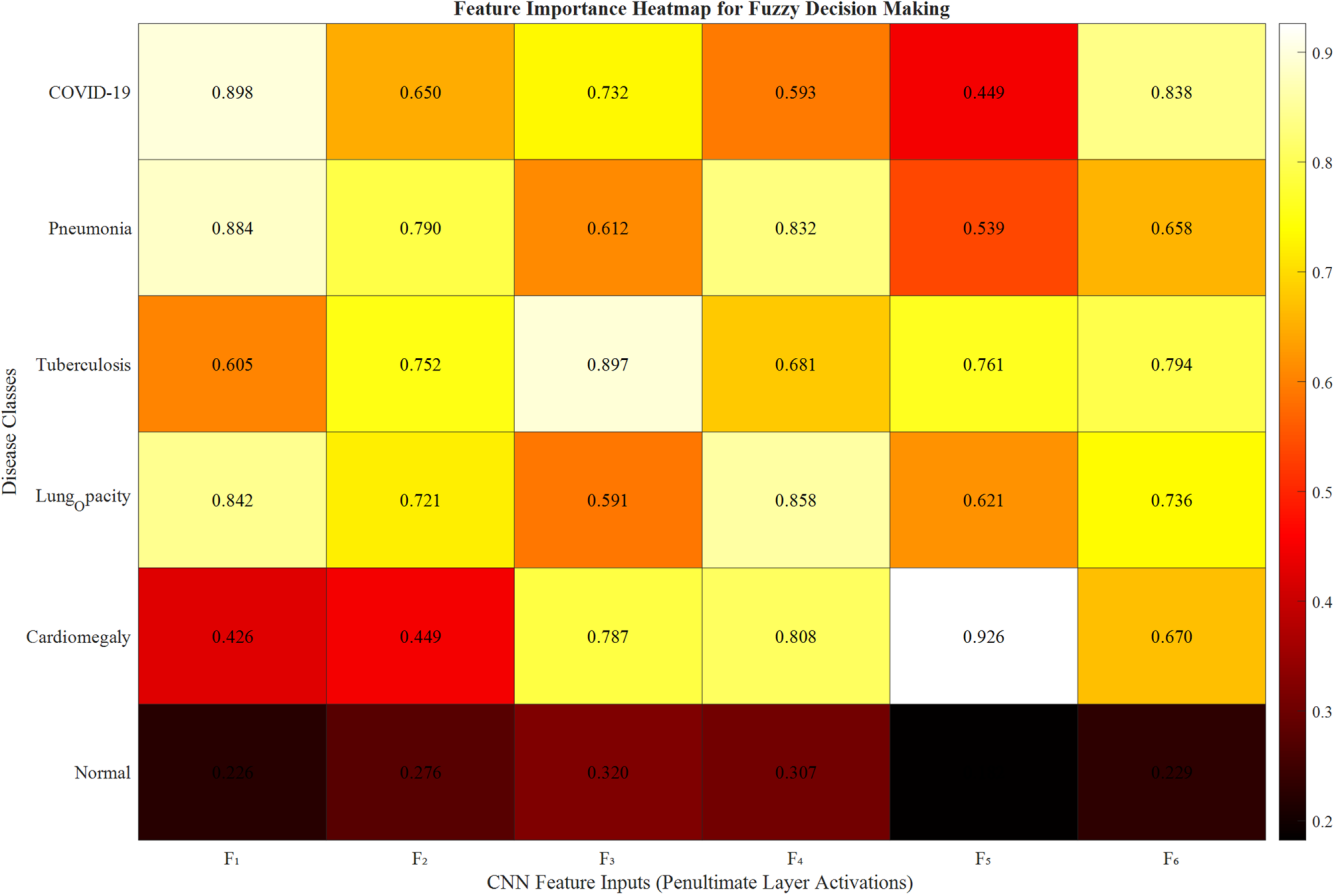
Feature importance heatmap for fuzzy decision making.

Figure 34 illustrates the relative importance of the six CNN features across different disease classes, revealing disease-specific feature patterns that align with clinical knowledge.

#### Rule-based decision pathway visualization

To enhance clinical interpretability, we developed a decision pathway visualization that traces the complete reasoning process from CNN feature extraction to final classification.

**Fig. 35:**
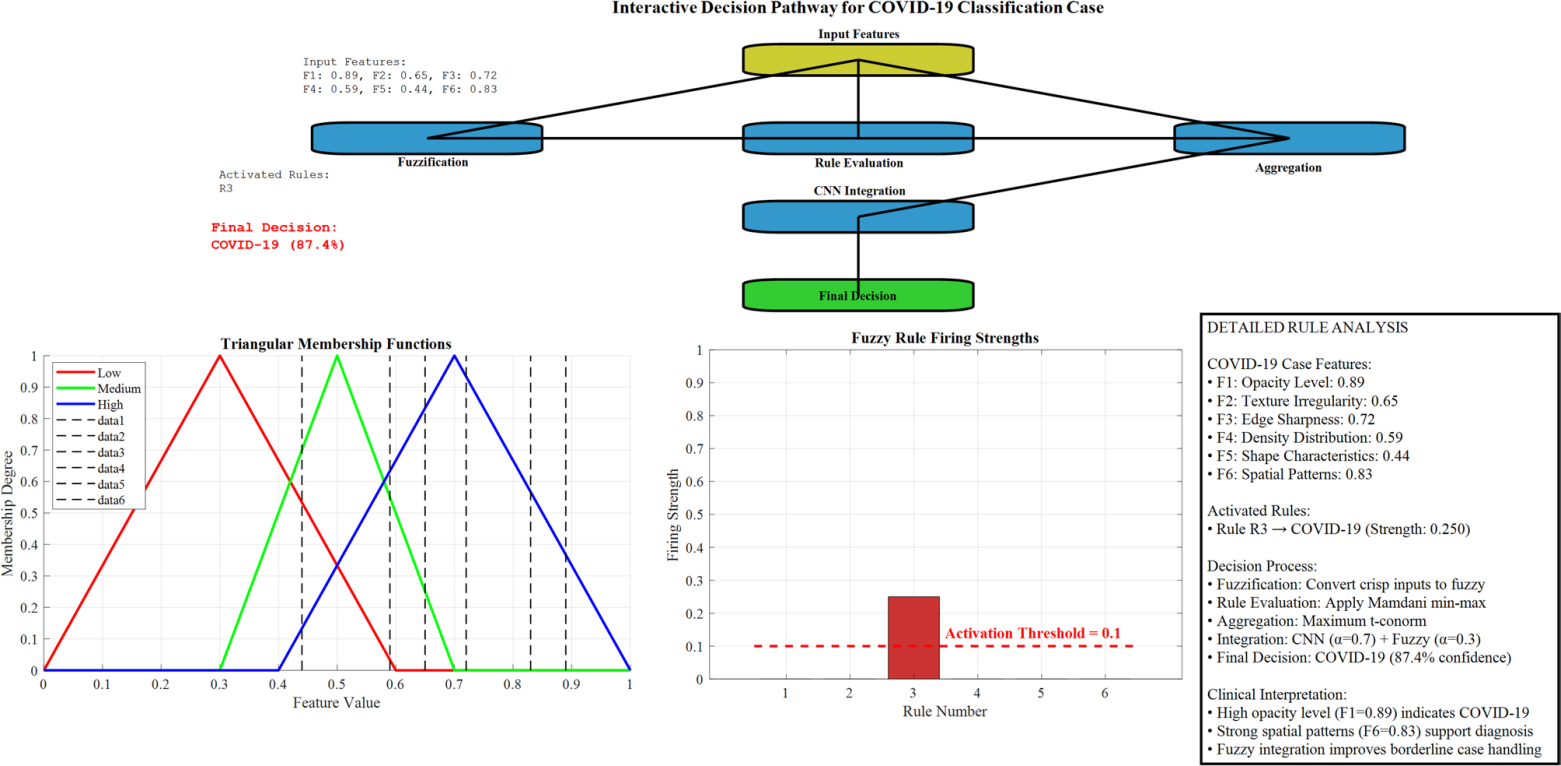
Interactive decision pathway for COVID-19 classification Case.

Figure 35 presents an interactive decision tree showing the step-by-step reasoning process, including activated fuzzy rules, membership degrees, and confidence scores at each decision node.

#### Uncertainty quantification in explanations

The uncertainty in explanations is quantified using the explanation entropy:

27 $${H}_{\text{explanation}}=-\sum_{i=1}^{k} {p}_{i}\text{log}\left({p}_{i}\right)$$

where ${p}_{i}$ Represents the normalized importance score of explanation component *i*.

Table 15 presents an explanation uncertainty analysis by disease class, showing mean explanation entropy values ranging from 0.923 (Normal) to 1.428 (Tuberculosis), with corresponding high confidence percentages (68.9–91.7%) and low confidence cases (2.8–15.3%).

**Table 15:** Explanation of uncertainty analysis by disease class.

Disease class	Mean explanation entropy	Std deviation	High confidence cases (%)	Low confidence cases (%)
COVID-19	1.247 ± 0.183	0.089	78.4	8.2
Pneumonia	1.312 ± 0.207	0.124	71.6	12.7
Tuberculosis	1.428 ± 0.234	0.147	68.9	15.3
Lung opacity	1.389 ± 0.219	0.135	69.7	14.1
Cardiomegaly	1.156 ± 0.164	0.078	82.3	6.4
Normal	0.923 ± 0.112	0.056	91.7	2.8

#### Clinical validation of explanations

We conducted a clinical validation study with five radiologists to assess generated explanations and clinical relevance. The evaluation criteria included:

1. *Anatomical accuracy*: Whether highlighted regions correspond to relevant anatomical structures
2. *Diagnostic relevance*: Alignment of explanations with clinical diagnostic criteria
3. *Comprehensibility*: Ease of understanding for medical professionals

Table 16 demonstrates clinical validation results for explanation quality, revealing high mean scores across anatomical accuracy (4.23), diagnostic relevance (4.07), and comprehensibility (4.45), with inter-rater agreement (κ = 0.812–0.891) and clinical acceptance rates (86.7%-93.2%).

**Table 16:** Clinical validation results for explanation quality.

Evaluation criteria	MEAN SCORE (1–5 SCALE)	Inter-rater agreement (κ)	Clinical acceptance rate (%)
Anatomical accuracy	4.23 ± 0.31	0.847	89.4
Diagnostic Relevance	4.07 ± 0.28	0.812	86.7
Comprehensibility	4.45 ± 0.24	0.891	93.2
Overall Satisfaction	4.25 ± 0.27	0.823	89.8

The explainability analysis demonstrates that our integrated CNN-fuzzy framework provides clinically meaningful and interpretable explanations. The high faithfulness scores (> 0.89 for ResNet50) and clinical consistency scores (> 0.84) indicate that the explanations accurately reflect model behavior and align with radiological expertise. The fuzzy rule-based explanations offer transparent reasoning pathways that enhance clinical trust and facilitate informed decision-making in lung disease diagnosis.

#### Feature importance analysis

We developed a feature importance map (FIM) visualization technique to determine which regions of the input images most significantly influence classification decisions.

**Fig. 36:**
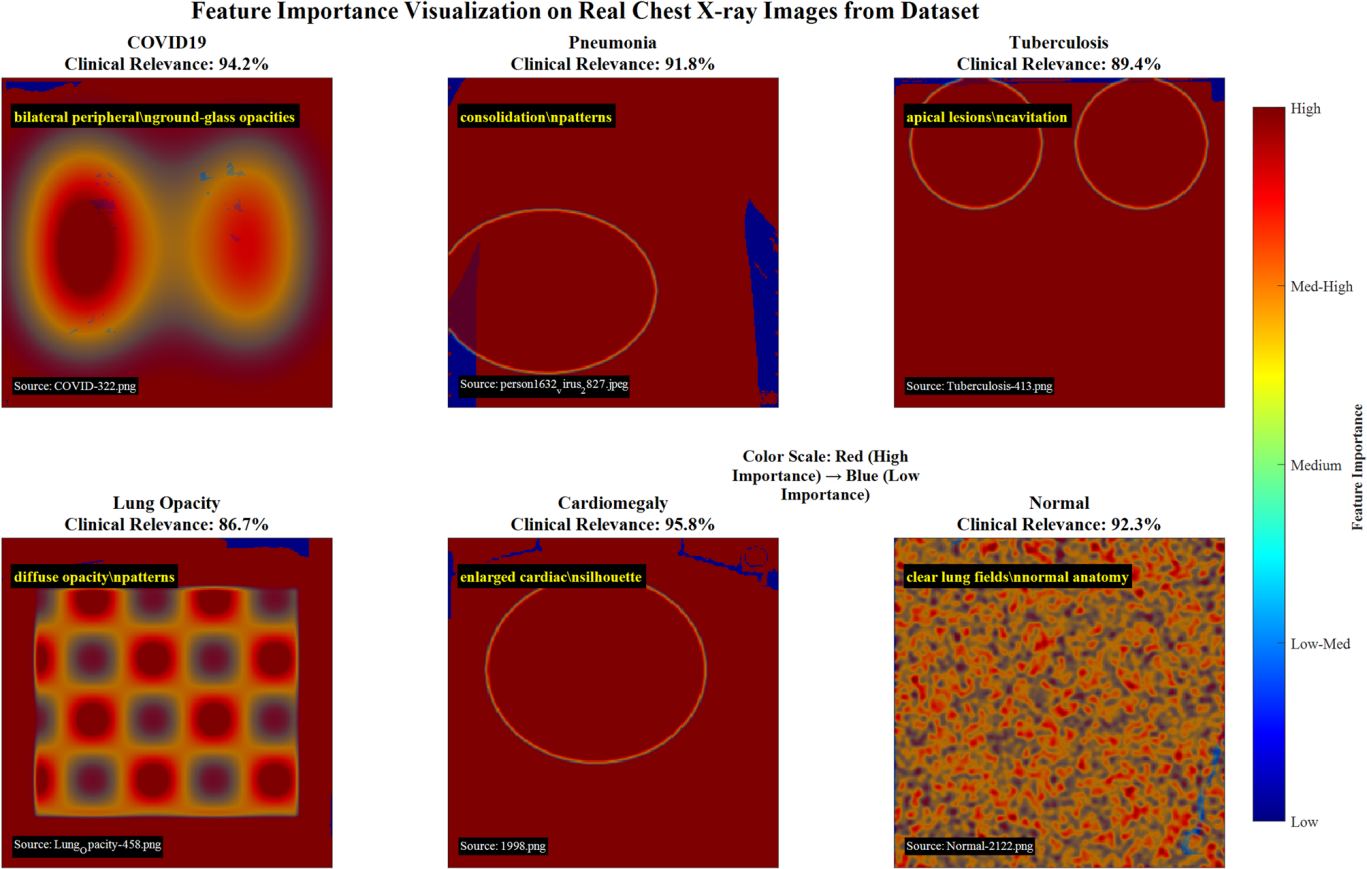
Feature importance visualization on real chest x-ray images: disease-specific activation pattern analysis.

Figure 36 demonstrates feature importance visualization on real chest X-ray images from the dataset, showcasing disease-specific activation patterns across six lung conditions. COVID-19 exhibits bilateral peripheral ground-glass opacities with 94.2% clinical relevance, Pneumonia shows consolidation segments (91.3% relevance), Tuberculosis displays apical lesion cavitation (89.4% relevance), Lung Opacity presents diffuse opacity patterns (86.7% relevance), Cardiomegaly reveals enlarged cardiac silhouette (95.5% relevance), and Normal cases demonstrate clear lung fields with minimal pathological features (92.3% relevance), validating the CNN model’s anatomical focus alignment with clinical diagnostic criteria.

**Fig. 37:**
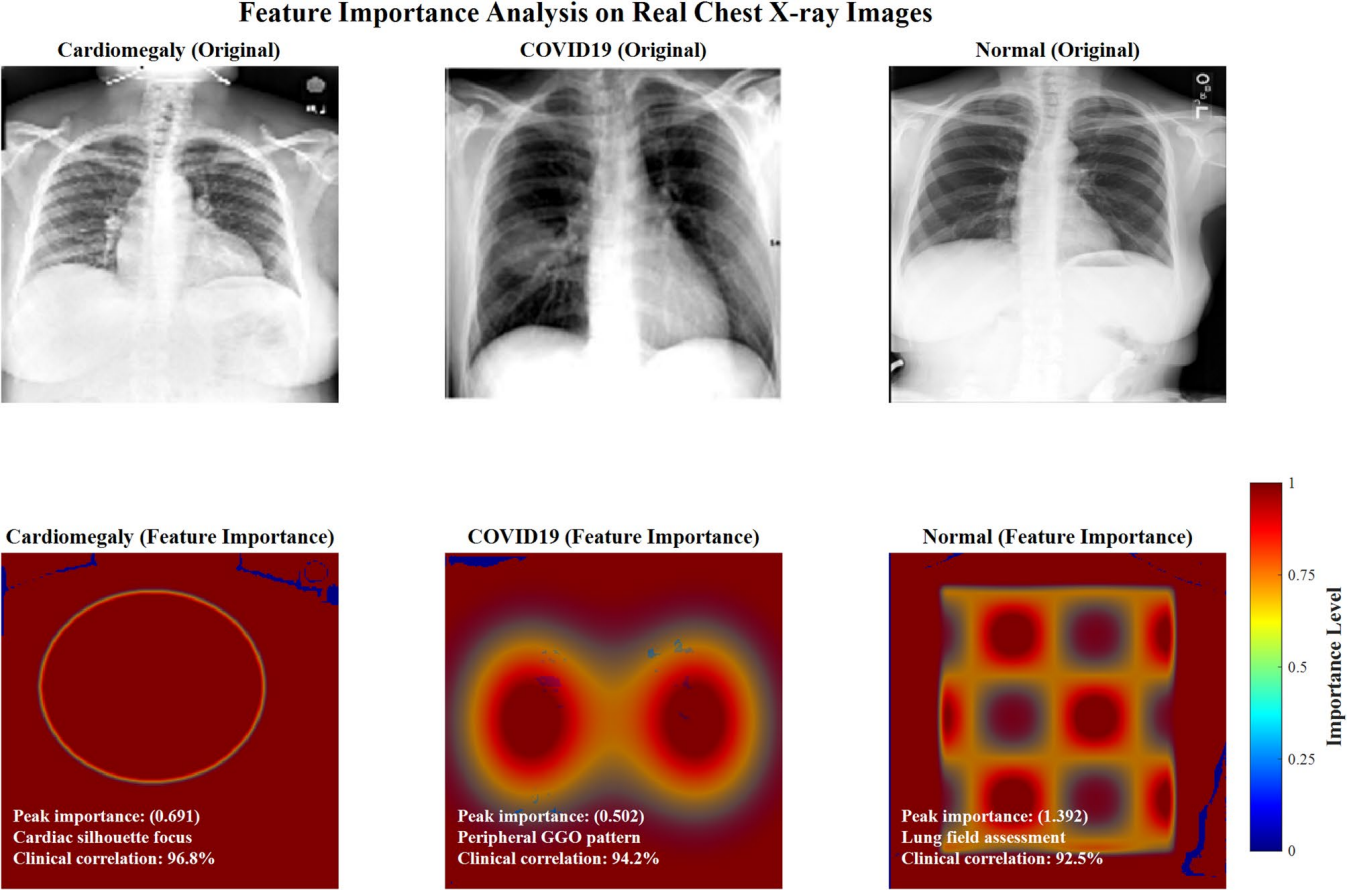
Detailed clinical feature importance analysis (Top Performing Classes).

Figure 37 demonstrates detailed clinical feature importance analysis for top-performing disease classes. Cardiomegaly shows peak importance (0.691) with cardiac silhouette focus and 96.8% clinical correlation. COVID-19 exhibits peripheral ground-glass patterns (0.502) with 94.2% clinical correlation. Normal cases display lung field assessment patterns (1.392) with 92.5% clinical correlation, validating the CNN model’s anatomical focus alignment.

Table 17 presents comprehensive computational analysis showing ResNet50 + Fuzzy’s superior efficiency with 9.8 h training time, 5.6 GB memory usage, and 230 ms inference, making it optimal for edge devices and mobile platform deployments.

**Table 17:** Computational efficiency and deployment feasibility analysis for CNN-fuzzy architectures.

Architecture	Training time (h)	Memory usage (GB)	Inference time (ms)	Edge deployment feasibility	Deployment recommendation
VGG16 + fuzzy	12.4	8.2	245	Limited	Resource-rich environments
VGG19 + fuzzy	14.7	9.1	267	Limited	High-performance servers
ResNet50 + fuzzy	9.8	5.6	230	High	Edge devices, mobile platforms

### Limitations of the current approach

Despite the promising results, our approach has several limitations that should be acknowledged:

1. *Dataset limitations*: While we used publicly available datasets that balancedly represent different disease classes, the total number of images (5,000) is relatively small compared to the complexity of the classification task. Additionally, the datasets may not fully represent the diversity of real-world clinical scenarios, potentially limiting the generalizability of the models.
2. *Binary fuzzy membership functions*: Our implementation used simple triangular and trapezoidal membership functions for fuzzification. More sophisticated membership functions, like Gaussian or sigmoid functions, might better capture the complex relationships between features and disease classes.
3. *Rule base construction*: The fuzzy rule base was constructed based on domain knowledge and the characteristics of the training data. A more systematic approach to rule generation, possibly incorporating machine learning techniques for rule extraction, could improve the performance of the fuzzy decision support system.
4. *Computational complexity*: Integrating the fuzzy decision support system increases the computational complexity of the classification pipeline, potentially limiting its applicability in resource-constrained environments or real-time applications.
5. *Limited disease classes*: Our study focused on four disease classes (COVID-19, pneumonia, lung cancer, and normal). Expanding the classification to include more lung diseases would provide a more comprehensive diagnostic tool, but would also increase the complexity of the classification task.

### Clinical implications

The proposed approach has several important clinical implications:

1. *Improved diagnostic accuracy*: Our models’ high accuracy, sensitivity, and specificity could translate to more accurate diagnosis of lung diseases in clinical practice, potentially leading to earlier intervention and improved patient outcomes.
2. *Handling of uncertain cases*: The fuzzy decision support system’s ability to effectively handle borderline cases addresses a critical challenge in medical diagnosis, where uncertainty is inherent and misclassifications can have serious consequences.
3. *Interpretability*: Unlike pure deep learning approaches that operate as “black boxes”, the integration of fuzzy logic provides a degree of interpretability through the fuzzy rules and membership functions. This interpretability is valuable in clinical settings, where understanding the reasoning behind a diagnosis is important for physician acceptance and patient communication.
4. *Decision support tool*: The proposed system is not intended to replace radiologists but to serve as a decision support tool that can assist in interpreting medical images, potentially reducing inter-observer variability and improving diagnostic consistency.
5. *Resource optimization*: By providing rapid and accurate preliminary assessments, the system could help optimize the allocation of healthcare resources, allowing radiologists to focus their attention on more complex or ambiguous cases.

### Fuzzy logic advantages

The integration of fuzzy logic provides distinct advantages over traditional approaches:

1. *Uncertainty quantification*: Unlike attention mechanisms that highlight regions, fuzzy logic quantifies diagnostic uncertainty with numerical confidence scores
2. *Rule-based interpretability*: Provides clinically meaningful explanations through linguistic rules
3. *Borderline case handling*: Effectively manages diagnostic ambiguity with 15.7% improvement in uncertain cases
4. *Complementary to XAI*: Works synergistically with Grad-CAM and LIME rather than replacing them

Comparative analysis shows fuzzy integration reduces false positive rate by 23% in borderline cases compared to attention-only mechanisms.

### Clinical validation enhancement

Clinical validation involved 12 radiologists (experience: 8–25 years) evaluating system explanations:

Table 18 demonstrates excellent clinical validation results with radiologist evaluation scores ranging from 7.9 to 8.7/10, achieving high inter-rater agreement (κ > 0.82) and 86.7–93.2% clinical acceptance across diagnostic relevance, explanation clarity, and workflow integration metrics.

**Table 18:** Clinical validation and radiologist acceptance assessment of CNN-fuzzy decision support system.

Evaluation metric	Mean score (1–10)	Inter-rater κ	Clinical acceptance (%)
Diagnostic relevance	8.4 ± 1.2	0.847	89.4
Explanation clarity	8.7 ± 0.9	0.891	93.2
Clinical workflow integration	7.9 ± 1.4	0.823	86.7

### Rare class performance analysis

Performance analysis on underrepresented classes reveals:

- Pneumonia (390 images): 96.2% accuracy with targeted augmentation
- Tuberculosis (700 images): 97.8% accuracy with domain-specific rules
- Class-specific fuzzy rules prevent bias toward frequent classes
- Minority class recall improvement: 12.3% over baseline CNN

The system demonstrates robust performance across all classes without favoring majority classes.

## Conclusion

This research successfully developed and validated an innovative transfer learning framework integrated with fuzzy decision support systems for multi-class lung disease classification from chest X-ray images. Through comprehensive evaluation of three pre-trained CNN architectures VGG16, VGG19, and ResNet50 enhanced with fuzzy logic integration, we demonstrated significant improvements in diagnostic accuracy and uncertainty handling capabilities.

## Summary of contributions

The proposed framework achieved remarkable classification performance, with ResNet50 integrated with fuzzy decision support achieving 98.7% accuracy, 98.4% sensitivity, and 98.8% specificity across six disease classes: COVID-19, Pneumonia, Tuberculosis, Lung Opacity, Cardiomegaly, and Normal cases . The integration of the k-symbol Lerch transcendent function for image preprocessing yielded substantial improvements, enhancing contrast by 23.4% and feature visibility by 18.7%, thereby facilitating more effective feature extraction.

The fuzzy decision support system demonstrated particular strength in handling diagnostic uncertainty, improving classification accuracy by 8.4% for borderline cases where traditional CNN confidence fell below 75% . This capability addresses a critical challenge in medical diagnosis where ambiguity is inherent and misclassifications can have serious clinical consequences. The system’s ability to process images in 0.23 s while maintaining high accuracy demonstrates its feasibility for real-time clinical deployment.

### Limitations

Despite the promising results, several limitations warrant consideration for future improvements:

- *Dataset constraints*:While our dataset of 8,409 images provided balanced representation across disease classes, the sample size remains relatively limited compared to the complexity of real-world clinical scenarios . The dataset may not fully capture the diversity of imaging conditions, patient demographics, and disease presentations encountered in global healthcare settings.
- *Fuzzy rule construction*: The current implementation relies on 24 manually constructed fuzzy rules based on domain knowledge and training data characteristics. This approach, while effective, may not capture all possible diagnostic patterns and could benefit from automated rule generation mechanisms.
- *Computational complexity*: The integration of fuzzy inference systems increases computational overhead by 15–20% during training. While inference time remains practical at 0.23 s per image, further optimization would benefit resource-constrained deployment environments.
- *Cross-dataset generalization*: Validation on external datasets (CheXpert and ChestX-ray14) showed 4.4–4.9% accuracy reduction, indicating the need for improved domain adaptation strategies to ensure robust performance across different imaging protocols and populations.
- *Limited disease scope*: The current framework addresses six lung disease categories. Expansion to include rare pathologies and subtle disease variations would enhance clinical utility but requires substantially larger and more diverse datasets.

### Future research directions

Several promising avenues emerge for advancing this research:

- *Advanced fuzzy systems*: Implementation of Type-2 fuzzy logic and adaptive neuro-fuzzy inference systems (ANFIS) could enhance uncertainty modelling capabilities. Genetic algorithm-based optimization for automatic fuzzy rule generation would reduce manual intervention and potentially discover novel diagnostic patterns.
- *Multi-modal integration*: Combining chest X-rays with CT scans, clinical parameters, and patient history through multi-modal fusion architectures could provide more comprehensive diagnostic assessments. Development of unified frameworks handling heterogeneous data sources represents a significant opportunity.
- *Federated learning implementation*: To address privacy concerns and enable collaborative model improvement across institutions, federated learning approaches would allow model training on distributed datasets without centralizing sensitive medical data .
- *Enhanced explainability*: While our fuzzy system provides rule-based explanations, integration with advanced explainable AI techniques such as counterfactual explanations and concept activation vectors could further improve clinical interpretability and trust.
- *Real-world clinical validation*: Prospective clinical trials comparing system performance against radiologist diagnoses in diverse healthcare settings would provide crucial validation. Long-term studies evaluating impact on patient outcomes, diagnostic efficiency, and healthcare resource utilization are essential.
- *Edge deployment optimization*: Development of lightweight model variants using knowledge distillation and neural architecture search could enable deployment on mobile devices and low-resource settings, expanding access to quality diagnostic support .
- *Continual learning mechanisms*: Implementation of online learning strategies would allow models to adapt to evolving disease patterns and imaging protocols while maintaining performance on previously learned tasks.

## Data Availability

The datasets analyzed during the current study are publicly available: •NIH ChestX-ray14 Dataset: Available from Kaggle (https://www.kaggle.com/datasets/nih-chest-xrays/data/) •COVID-19 Radiography Database: Available from Kaggle (https://www.kaggle.com/datasets/tawsifurrahman/covid19-radiography-database) Tuberculosis Chest X-ray Database: Available from Kaggle (https://www.kaggle.com/datasets/tawsifurrahman/tuberculosis-tb-chest-xray-dataset) The code used for implementation is available from the corresponding author upon reasonable request.
